# Integrated Analysis of Residue Coevolution and Protein Structure in ABC Transporters

**DOI:** 10.1371/journal.pone.0036546

**Published:** 2012-05-08

**Authors:** Attila Gulyás-Kovács

**Affiliations:** Laboratory of Cardiac/Membrane Physiology, Rockefeller University, New York, New York, United States of America; Georgia Institute of Technology, United States of America

## Abstract

Intraprotein side chain contacts can couple the evolutionary process of amino acid substitution at one position to that at another. This coupling, known as residue coevolution, may vary in strength. Conserved contacts thus not only define 3-dimensional protein structure, but also indicate which residue-residue interactions are crucial to a protein’s function. Therefore, prediction of strongly coevolving residue-pairs helps clarify molecular mechanisms underlying function. Previously, various coevolution detectors have been employed separately to predict these pairs purely from multiple sequence alignments, while disregarding available structural information. This study introduces an integrative framework that improves the accuracy of such predictions, relative to previous approaches, by combining multiple coevolution detectors and incorporating structural contact information. This framework is applied to the ABC-B and ABC-C transporter families, which include the drug exporter P-glycoprotein involved in multidrug resistance of cancer cells, as well as the CFTR chloride channel linked to cystic fibrosis disease. The predicted coevolving pairs are further analyzed based on conformational changes inferred from outward- and inward-facing transporter structures. The analysis suggests that some pairs coevolved to directly regulate conformational changes of the alternating-access transport mechanism, while others to stabilize rigid-body-like components of the protein structure. Moreover, some identified pairs correspond to residues previously implicated in cystic fibrosis.

## Introduction

The increasing number of solved protein structures raises the question how structural data can help clarify the biochemical mechanisms underlying protein function. Although extremely informative, even the complete map of residue contacts is in general insufficient to reveal biochemical mechanisms. Experiments mutating specific amino acid positions are essential complements to structure but the typically low throughput of these experiments calls for highly specific, rational design. Sometimes structural models themselves highlight experimental candidate positions but more often additional information is needed. This is especially so when specific functional interactions, represented by pairs of positions, are to be tested [1], [2] since the number of candidate pairs scales, in principle, as the square of the number of candidate positions.

The superfamily of ATP-binding cassette (ABC) transporters is an epitome of proteins with recently determined structures but poorly understood biochemical mechanisms [3], [4]. Their members actively transport substrate molecules across membranes with the exception of the (passive) ion channel CFTR (a member of the ABC-C family), whose defect causes cystic fibrosis disease. Typical members of the ABC-B and ABC-C families are active exporters, like the MDR and MRP proteins (notably Pgp/MDR1), which recognize anticancer drugs as their natural substrates and thereby confer multidrug resistance on tumor cells.

**Figure 1:**
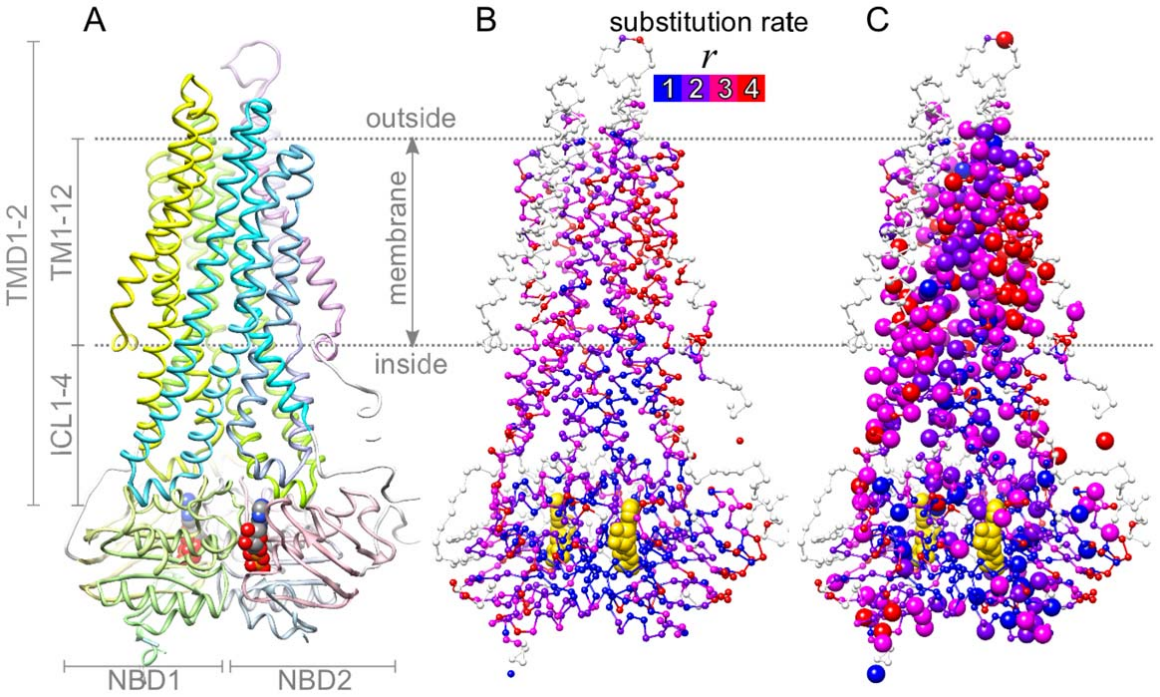
Structure of ABC-C proteins and the rate of amino acid substitution. (**A-C**) Homology model of the ABC-C protein CFTR [45]. (**A**) Main structural components. *NBD*: nucleotide binding domain; *TMD*: transmembrane domain; *TM*: transmembrane helix; *ICL*: intracellular loop. ATP-molecule atoms are shown as spheres. (**B**) Each amino acid position 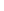 is marked by a small sphere at the C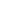 atom and is colored according to 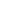, the estimated discretized substitution rate (eq. 21). 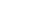 (blue) indicates that 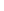 is conserved. (**C**) The large spheres represent the set of positions predicted in this study to coevolve with some other position(s) in the same set. Structural figures were made using UCSF Chimera [70].

All ABC-B and ABC-C transporters are built of two transmembrane domains (TMDs), which interact directly with the translocating substrate, and two nucleotide binding domains (NBDs), which convert chemical to mechanical energy by binding and hydrolyzing ATP (Figure 1A). The popular alternating-access transport model asserts that this mechanical energy drives a conformational cycle coupled to unidirectional transport, and during each cycle the TMDs alternate between inward and outward-facing conformation [5]. This model, although supported by relatively high-resolution structures [3], [4], describes transport mechanism at a resolution that is too low for the clarification of many crucial details related to multidrug resistance or cystic fibrosis. For a refined model, mechanistically crucial residue-residue interactions need to be somehow predicted and experimentally tested: particularly between the transmembrane helices (TM1,TM12), which are relatively understudied, and whose extensions form intracellular loops (ICL1,ICL4), which couple the TMDs to the NBDs (Figure 1A).

The abundance of sequenced ABC-B and ABC-C proteins makes these families ideal for comparative sequence analysis. Such analysis can infer those structural and functional constraints on sequence evolution that are not necessarily evident from sole structural analysis. For example, side chain contacts can couple the process of amino acid substitution at one position to that at the contacting position and thereby induce residue coevolution, but the strength of coupling and its persistence in time may vary [6], [7]. Therefore, statistical techniques predicting coevolving pairs, henceforth referred to as coevolution detectors, have been utilized for different purposes. When the representative structure of some protein family is unknown, then coevolution detectors can be used to predict contacts and thereby aid structure determination [8]–[16]. But when such structure is known, detectors are still useful for the prediction of the subset of contact pairs that exhibit strong and permanent coevolution [11], [17]–[25]. The latter set of pairs can be interpreted as a representation of conserved and general mechanisms that characterize the whole protein family. Therefore, these pairs are highly relevant for the elucidation of these mechanisms as either self-standing results or pointers for the rational design of “double mutants” [1], [2], [26]–[28] for functional experiments.

All coevolution detectors predict coevolving pairs from multiple sequence alignments but they differ from each other in crucial assumptions on the substitution process, which can profoundly affect prediction accuracy. Yet the relative performance of individual detectors in accuracy tests remains unclear even after side by side comparison [29], [30], suggesting that accuracy strongly depends on the specific protein family and certain properties of the corresponding alignment. Therefore, a key question is: given a collection of detectors and a protein family with representative sequences and structure(s), how can coevolving pairs be detected the most accurately?

**Figure 2:**
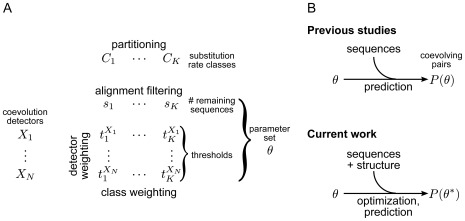
Integrative framework for the prediction of coevolving position pairs. (**A**) Parameters of the framework, and weighting and filtering procedures controlling them. *Partitioning* the set of all position pairs into substitution rate classes 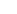 (eq. 10, 20–22), and *weighting* each *class* (eq. 11–13), addresses the sensitivity of coevolution detectors to substitution rate. *Detector weighting*: previous studies employed coevolution detectors 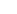 either separately or in a combination 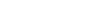 in which all 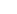 were equally weighted. However, equal weighting of 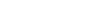 is not generally the optimal combination as demonstrated below in Figure 3B. The new framework allows unequal weighting of detectors (eq. 15). *Alignment filtering* (eq. 9, 14) removes redundant sequences from the input data (the sequence alignment) to minimize the adverse influence of phylogenetic redundancies on detectors. (**B**) Previous studies predicted coevolving position pairs in a protein family from only the corresponding sequence alignment, while ignoring useful information in solved structures. The current work makes use of structural information to adjust the parameters of detector weighting, class weighting and alignment filtering (parameter set 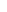) for optimal performance, as gauged by prediction of known structural contacts (eq. 5, 19).

The present study addresses that question with a new, integrative framework (Figure 2), which improves accuracy by directly incorporating structural information and by combining multiple detectors. Moreover, it features procedures that deal with the well-known vulnerability of detectors to the statistical non-independence of homologous sequences [31]–[33] and to the heterogeneity of positions with respect to substitution rate [34], [35]. This framework is employed to ABC-B and ABC-C transporters to predict those contact pairs that represent evolutionarily conserved interactions (i.e. coevolving pairs). The predicted pairs are presented with a particular attention to the possible mechanistic coupling between TM helices in both the inward and outward conformation of the TMDs.

## Methods

### Central Assumptions of the New Framework

Considering pairs of amino acid positions in a protein family, assume that, for each pair, the two positions either strongly and permanently coevolve with each other or evolve completely independently. Let 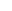 denote the set of *coevolving* pairs. Let 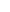 represent the set of (structural) *contact* pairs, specifically side chains contacts. Following pioneering studies [13], [14], [16] an intimate relationship has been conjectured between coevolution and side chain contact. The relationship can be stated in terms of the probabilities 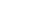 and 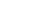 that, for some protein family, a random draw from all pairs or from contact pairs, respectively, gives a coevolving pair:
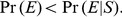
(1)This says that the contact pairs tend to be the coevolving pairs. Let 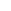 be the set of coevolving pairs *predicted* by some coevolution detector from sequence data 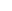. If the detector is useful then conditioning on 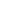 has similar effect to conditioning on 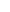:
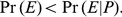
(2)Supporting the preceding two assertions it has been shown repeatedly [11]–[14], [16], [20], [22], [23], [29]–[32], [35]–[42] that most detectors can predict contact pairs better than random choice, and so
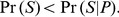
(3)Instead of predicting contact pairs to aid de novo prediction of structure, several studies [11], [18]–[25] aimed to detect coevolving pairs given the set of contact pairs assuming that



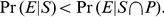
(4)The new framework was designed towards that aim and takes all above assumptions and findings as a starting point. As Figure 2 shows, 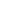 depends on a set of parameters 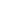, which specifies the identity of the detector (when a single detector is used) or the relative weights of detectors (when multiple detectors are combined). 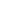 also determines how data are analyzed by a given (set of) detector(s): how classes of pairs are weighted and how the input alignment is filtered (Figure 2A). Therefore, if the protein structure is known, then 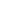 can be adjusted for optimal prediction of contact pairs. The individual parameters and the optimization problem will be precisely stated later; at this point another possible formulation is given to be consistent with eq. 3:
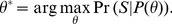
(5)A crucial assumption of this study is that the optimization in eq. 5 improves the detection of coevolving pairs within the set of contact pairs:



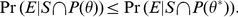
(6)Thus the central goal of this work is to find 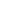, which uniquely determines 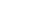 (Figure 2B) and ultimately 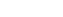. A key feature of the new framework is that the known structure plays a dual role in the current analysis. First, the structure is required for the optimization of the parameters (Eq. 5, Figure 2B bottom). Second, the structure (or some alternative conformation of that structure) is used to restrict the predicted pairs to the set of contact pairs by taking the intersection 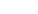 (Eq. 6).

### Parameters and Procedures of the New Framework

As mentioned above, 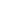 is a function of the parameter set 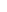. Now the question is: exactly what is 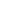, and how does it determine 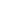 together with the data?

In general, a coevolution detector 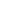 acts as a binary classifier that divides the set 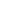 of all pairs into 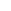 and the complementary set of pairs (the “negatives”). Given the input alignment data 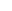, the condition for classification of each pair 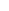 into 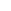 is that the test statistic 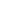 of the detector evaluated at 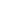 exceeds an adjustable threshold 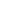:
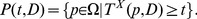
(7)It is practical to constrain the number of predicted pairs 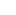 at some chosen fraction 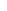 of all pairs by treating 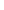 as a monotonically increasing function of 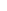. Then, for a given 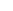 and 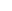,
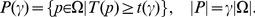
(8)Consequently, 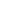 controls the true and false positive rate of the detector, which are defined subsequently in eq. 16–17.

The procedure of *filtering* of an alignment of homologous sequences, in particular *phylogenetic* type of filtering, aims to remove redundancies that emerge from the statistical non-independence within any collection of homologous sequences. These redundancies pose challenges to all coevolution detectors, especially to those assuming that homologous sequences are statistically independent from each other.

Any type of filter, applied to alignment 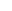, permutes sequences in a given order that depends on the filter type 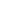. Then the filter removes a certain number of sequences in that order. Therefore, the filtered 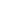 is determined both by 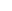 and by the number 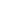 of sequences that remain in the alignment. It follows that, for a given 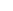,
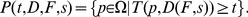
(9)Filtering will be discussed in more detail in Methods: Alignment Filtering.

For all detectors, 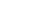 is known [34], [35], [38] to depend to some degree not only on the coevolution of position 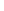 and 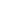 (where 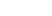) but also on the overall rate of amino acid substitution at 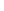 and at 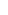. The dependence on substitution rate deteriorates the performance of the detector but can, in theory, be addressed by conditioning 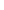 on the rates of the pair. Therefore, the new framework incorporates a novel strategy based on the procedure of *partitioning* 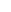 into 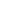 (substitution) *rate classes* 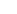 (Figure 2A):
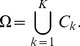
(10)The precise definition of 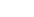 will be given later (eq. 20–22), but it may be worth emphasizing at this point that the members of each 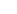 are position *pairs* and not single positions. Now a key feature of the new framework is that 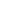 can be adjusted separately for each 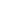 and that 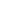 is defined as the union of the resulting 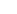
s:



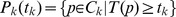
(11)

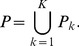
(12)


The vector 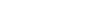 thus determines every 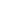 and therefore every 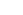. Like its scalar analog 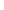, 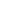 is also a function of 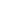, which imposes the constraint
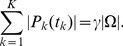
(13)(This is the same as the constraint expressed by the second equality in eq. 8, since 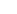
s are disjoint sets and thus 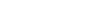.) The constraint in eq. 13 still allows individual 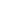
s to vary, which changes the relative size (the weights) of 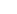
s. In this work the procedure of changing 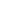, while requiring eq. 13 to hold, is referred to as *class weighting* procedure.

Partitioning 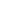 also allows the filtering of 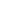 separately for each rate class so that there is a separate parameter 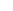 for each 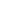,
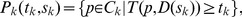
(14)and thus 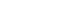 also depends on the vector 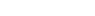. Eq. 14 corresponds to the combination of *partitioning + class weighting + filtering* in case of a general 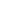 satisfying eq. 8, or to the combination of *partitioning + filtering* when all 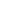
s are set to the same value. Note that in this case “combination” refers to *procedures* and not *detectors*.

**Figure 3:**
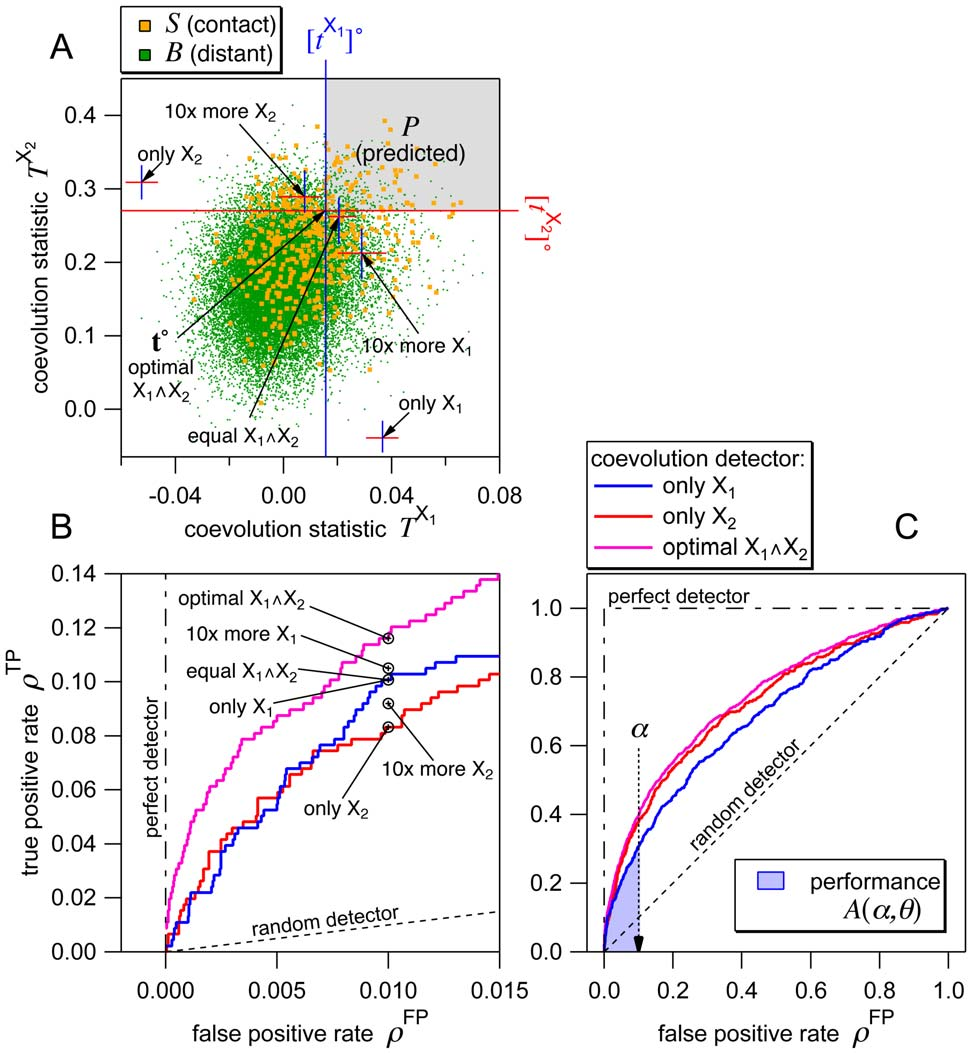
Weighted combination of coevolution detector 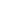 and 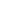. (**A**) Green and orange dots represent a set 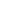 of pairs 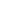 of amino acid positions in a protein family. 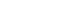, where 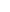 is the set of structural contact pairs (orange) and 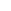 is the set of structurally distant pairs (green). 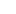 and 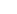 are coevolution detectors with statistics 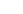 and 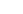, respectively, which are evaluated separately for each pair. A combined detector 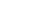 uses a pair of thresholds 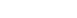 to define the set of predicted pairs 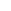 (eq. 15). The set of true positives is defined as 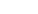; the true positive rate 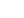 is linearly related to the number of true positives. False positives and the false positive rate 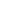 are defined analogously but with 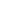 instead of 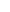 (eq. 16–17). Even if 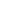 is fixed, 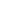 (and thus 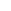) can still vary if 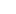 and 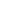 change in the opposite direction. Changing 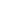 at fixed 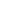 is called *detector weighting*. For example, 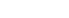 for all 6 thresholds 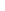 marked by the arrowheads. For the threshold labeled as “equal 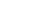
” the two detectors are combined in equal weights. “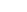 more 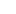
” refers to the weight of 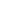 relative to 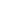. “Only 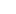
” means that 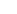 has zero weight and therefore 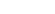 is the same as using 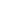 only. “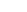 more 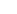
” and “only 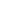
” have analogous meanings. Finally, the threshold denoted as 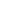 characterizes the optimally weighted 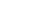, which by definition has the highest 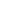 for each 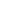. Black circles in (**B**) indicate 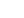 for all 6 thresholds, at 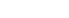, and thus report on the corresponding performance. The optimal 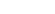 clearly outperforms the equally weighted one, which in this case happens to perform precisely as well as “only 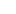 (their circles overlap). (**B**-**C**) Obtaining 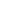 for all 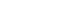 results in receiver operating characteristic curves, which describe the performance of coevolution detectors with respect to theoretical random, and perfect, detectors. Each curve is determined by the parameter set 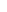, which includes 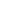 and therefore the weights on combined detectors. Integrating a curve on 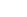 yields the area 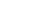, which is used as a scalar measure of performance (eq. 18, Figure 4, 5). Conditions: 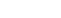; 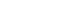; 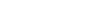; protein family  =  ABC-C; optimal phylogenetic filtering.

Up to this point a single detector 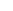 was assumed. Now let 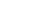 be a collection of 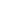 detectors, and let 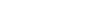 denote their logical AND combination [43] and 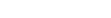 the corresponding thresholds (Figure 3A). Then the set of pairs predicted by the combined detector 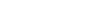 is defined as
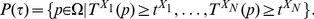
(15)It is clear that 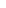 uniquely determines 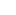 and that, for a given 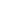, the constraint 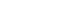 allows individual 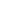
s to vary. For some 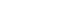, the impact of 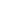 on 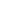, relative to that of any other detector 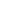 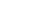, increases with 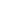. In other words, the weight of 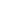 increases in 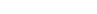. Therefore, adjusting 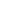
s relative to each other is referred to as the procedure of *detector weighting* and is illustrated by Figure 3A.

**Figure 4:**
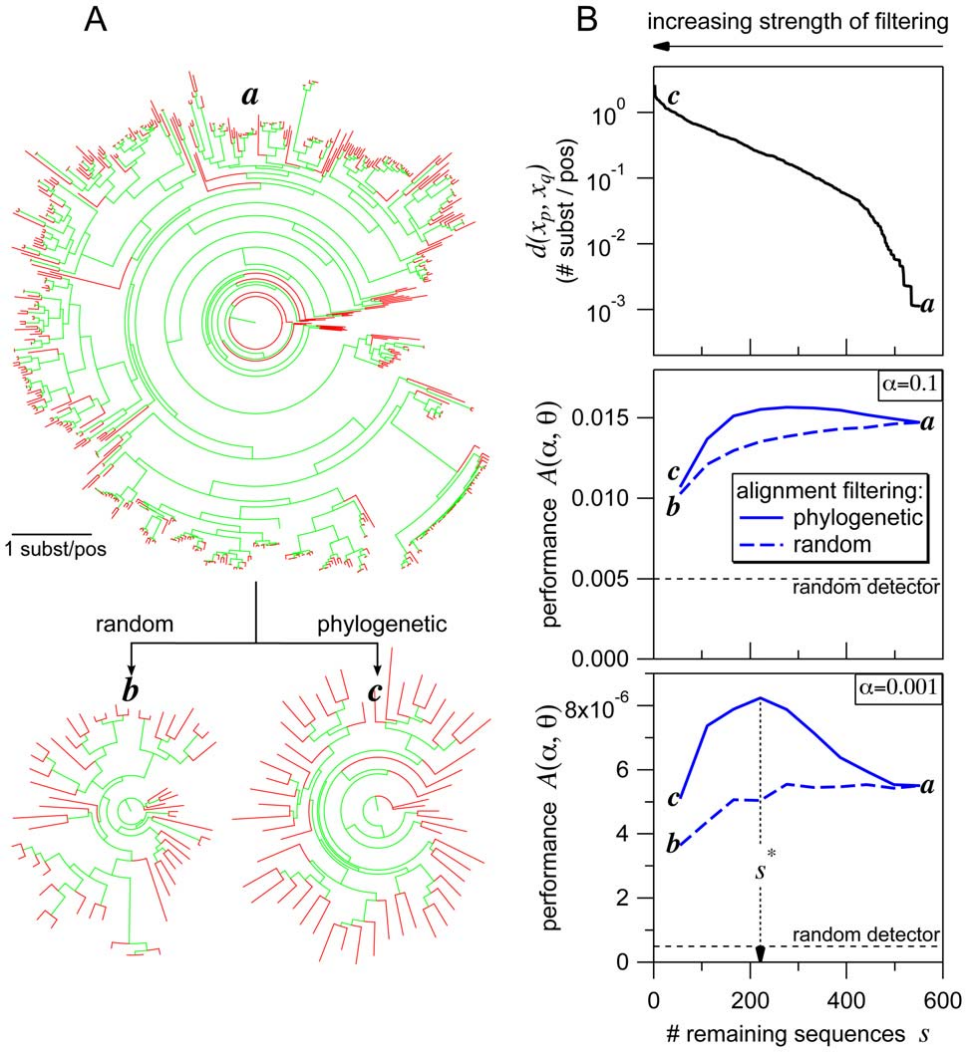
Influence of alignment filtering. (**A**) Random filtering and phylogenetic filtering both remove sequences from the unfiltered alignment, which is represented by the large tree ***a***, but result in trees (***b*** and ***c***) that differ in the length of terminal branches (red). Tree ***b*** (random filter) is similar to ***a*** in containing many extremely short terminal branches that are known to challenge coevolution detectors. In contrast, tree ***c*** (phylogenetic filter) lacks short terminal branches. (**B**) Opposing effects of progressively *increasing* strength of filtering, which leaves gradually *fewer* sequences in the alignment. The top graph shows, for the phylogenetic filter, the minimal sequence-sequence distance 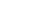 among all sequence pairs in the filtered alignment. The two lower graphs show performance, measured by 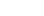, of a coevolution detector for both the phylogenetic and random filter. The first effect, specific to the phylogenetic filter, is a rise of 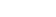 with *increasing* strength of filtering (*decreasing* number remaining sequences). This reflects the disappearance of short terminal branches, which in turn improves performance, until a maximum is reached around 250 sequences remaining. The second effect is the deterioration of performance with increasing strength of filtering, since fewer sequences provide less information for the coevolution detector. This effect is clearly seen for the random filter regardless of the number of remaining sequences but it becomes apparent for the phylogenetic filter only with strong filtering. Conditions: detector  =  MIp; protein family  =  ABC-C. Trees were plotted using FigTree v1.3.1 (http://tree.bio.ed.ac.uk/software/figtree/).

**Figure 5:**
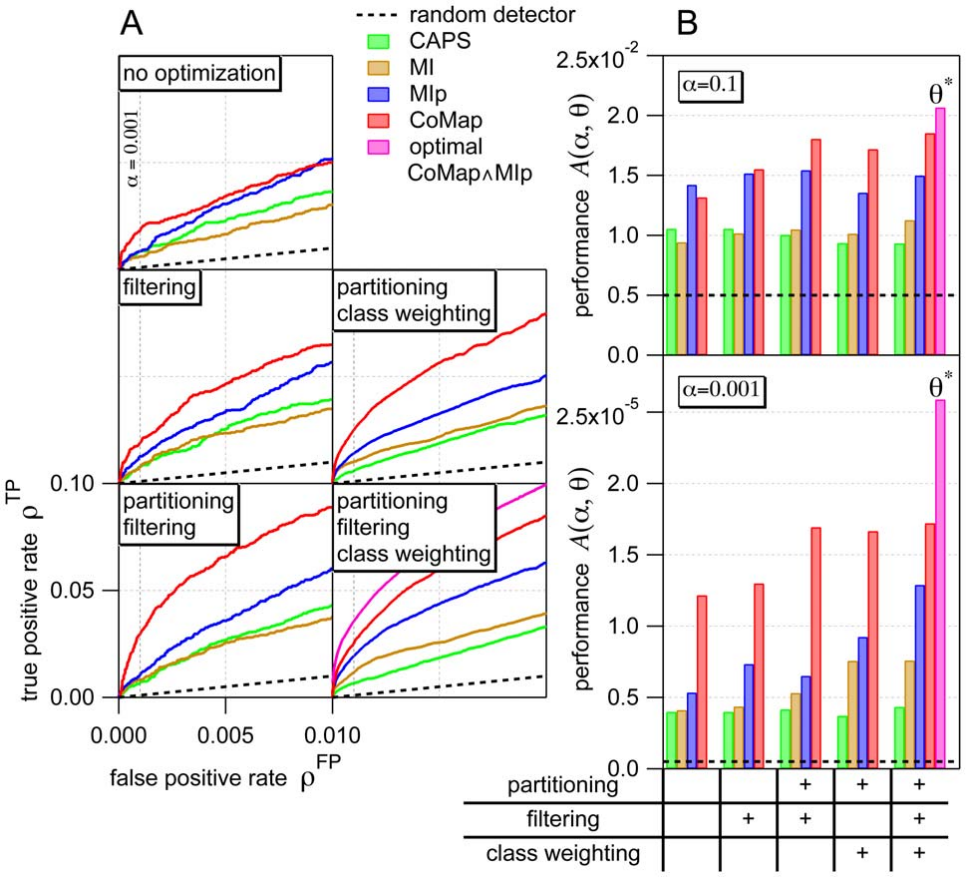
Optimizing the prediction of coevolving position pairs. Performance of several coevolution detectors (identified by color keys) characterized by (**A**) receiver operating characteristic curves and (**B**) partial area 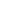 under these curves. Top graph in (**B**): low specificity (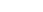); bottom graph: high specificity (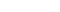). 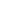 (above magenta bars) indicates the optimally weighted detector combination CoMap
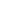
MIp after partitioning, optimal filtering and optimal class weighting (Figure 2). These optimal conditions yield the parameter set 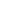 (eq. 19), which determines the set 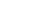 of predicted coevolving pairs, presented in Figure 6 and Table 2, 3. These results were obtained from the ABC-C dataset.

Given a specific detector 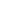, if 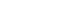 for all other detectors 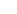 (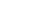), then the weight of these detectors vanish. This special case is equivalent to using detector 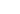 alone and not in combination with other 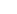
s. Furthermore, in the general case it is straight-forward to combine *detector weighting* with *partitioning + class weighting* (Figure 2A). Then each scalar 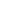 is replaced by a vector 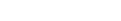 so that 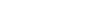. This can be further extended with *filtering*.

In summary, given the parameter 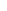, data 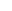, a filter type 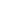, substitution rate classes 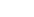 and a set 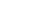 of detectors, the collection of parameters 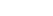 uniquely determines the set of predicted pairs 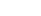 in the new framework. Next, it will be discussed how the optimal 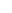 is actually found, and eq. 5 will be replaced by a closely related formula. This will be followed by detailed information on 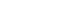 and 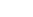.

### Optimization Using Structural Information

Let 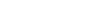 and 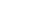 have the same meaning as before. Let 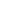 denote the set of contact pairs and 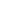 the set of pairs 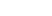 for which 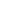 and 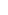 are separated by some substantial distance in 3D space, so that 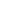 and 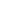 are unlikely to directly interact with each other in any native conformation of the protein. 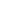 and 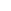 will be defined in the next subsection; for now assume that these sets are known. The true positive rate 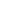 (sensitivity) and false positive rate 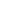 (reverse specificity) are defined, respectively, as
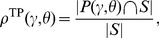
(16)

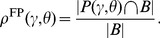
(17)As noted after eq. 8, 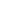 and 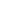 are functions of 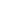, and therefore eq. 16–17, together with eq. 8, shows that 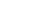 makes both 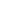 and 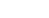. Likewise, 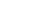 drives both 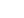 and 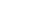. In general, 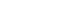 for a given detector and 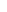. When 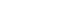, the detector is informative with respect to random selection. In contrast, for a theoretical *random detector* 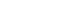 (Figure 3B-C, dashed line).

The receiver operator characteristic curve of a detector is a mapping that associates each 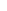 with 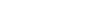 at a fixed 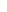 (Figure 3B-C). The partial area 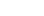 under the ROC curve is the Riemann-Stieltjes integral of 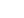 with respect to 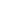 over the interval 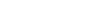:
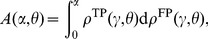
(18)Thus 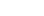 provides a scalar measure of performance at fixed 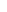 and 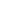. The interval 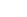 restricts 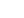 below a chosen 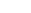. Small 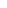 is desired when high specificity (obtaining low 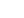) is more important than high sensitivity (achieving high 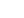), as in the case of this study. Note that 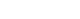 for a random detector.

Let 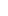 be a relation transforming 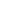 to 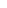 such that 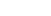. In the new framework, the optimal parameter set 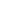 is defined as
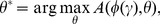
(19)replacing the initial formulation of the optimization problem (eq. 5). Thus, for each 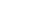, a unique 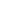 is obtained, which is precisely the central goal of this work (eq. 6).

In the present analysis of ABC transporters 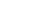 detectors 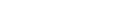 were employed, and 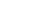 substitution rate classes 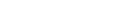 were used. This gave 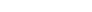 adjustable parameters 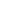 under the constraint expressed by eq. 13. In addition to this, filtering at separate 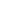 for each 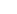 and 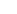 provided 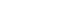 parameters and so the parameter space 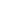 had a dimension of 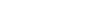. (Note that in Figure 2A the same 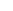 is used for all 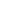.) To reduce 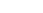, the present work employed a heuristic optimization strategy for eq. 19, whose details are described in Text S1 (see also Figure 3, S1 and S8).

### Structural Models and Contact Pairs

The set 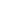 of contact pairs was defined as those pairs 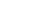 for which the distance 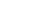 separating the C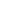 atom of position 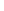 from that of 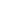 is less than 8Å in a structure representing the whole protein family. The set 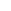 of distant pairs was defined by requiring 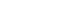
Å. The remaining “intermediate” pairs (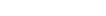
Å) were excluded from 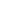 as in ref. [37] because a large fraction of them may be connected by chains of coevolving contact pairs [40], [42]. Thus 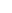 was obtained using only 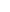 and 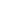. These sets were derived separately from Sav1866 (PDB: 2HYD) [44] and CFTR (homology model [45]) representing the ABC-B and the ABC-C family, respectively.


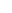
 includes the collection 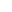 of optimized thresholds, which determines the set 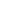 of predicted pairs (eq. 15). Next, a collection 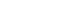 of sets of predicted contact pairs was obtained by using 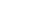, which was derived from a set of structures that correspond to distinct conformations of the same protein. For the ABC-B family, this set contained Pgp in the inward (3G5U [46]) and outward-facing [47] conformation, and for the ABC-C family, CFTR in the inward [48] and outward-facing [45] conformation. Consequently, a small fraction of predicted pairs were contact pairs selectively in some but not other conformations: for these pairs 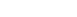 but 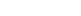 (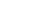).

### Amino Acid Substitution Model and Rate Classes

The definition of rate classes 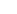 requires some discussion on the amino acid substitution model used in this study. The same model also played a role in the estimation of sequence-sequence distances (which were used for alignment filtering, as explained in the next subsection), in the inference of phylogenetic trees and in the evaluation of the coevolution statistic of certain detectors. Sequence-sequence distances and trees were both estimated by maximum likelihood using RAxML v7.0.4 [49].

The substitution of amino acid residues at each position was modeled as a continuous-time Markov process with a distinct transition rate between each pair of amino acids. The transition rates used in this study were those described by the WAG-F-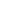 model [50]. In this model, the transition rates are scaled by a specific factor at each position 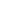; the scaling factor is known as the (overall) substitution rate 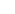. In other words, the substitution rate is allowed to vary among positions (p.110 of ref. [51]). Note that substitution rate is inversely related to “residue conservation”.

Considering all positions, the collection 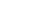 of rates is a set of independent, identically distributed random variables. The distribution is 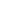-type with cumulative density function 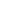. Given the number 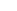 of rate classes of single positions a new random variable, the *discretized substitution rate* 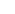, is defined as



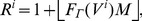
(20)where 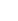 denotes the floor function. It follows directly from definition eq. 20 that 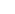 takes values on 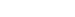 and has discrete uniform distribution with probability mass function 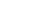 such that 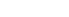 (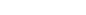).

This uniform “prior” probability mass function 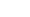 can be updated, for each position 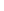, to the “posterior” the maximum likelihood estimate 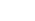 when an alignment and a tree is given. In this study this was done with CoMAP v1.3.0 [19] using the tree inferred from the alignment (which corresponds to an empirical Bayes approach; see p. 114 of ref. [51]). The *estimated* discretized substitution rate 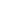 of position 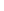 is defined as the mode of the posterior distribution 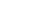:
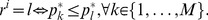
(21)


Given 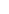 and 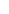 for each position pair 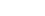, the class 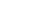 of pairs is defined as
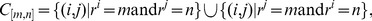
(22)where 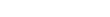. By the symmetry of the right side of eq. 22, 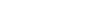 so it can be required that 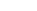. Then the number 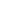 of classes of pairs is derived from 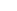 according to 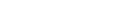. In this work 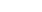 and so 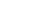 (Figure S2).

The notation 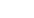 can be replaced by 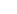 using any function that maps each 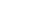 to a unique 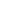. The present work uses the simpler 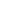 notation to refer to a rate class in general (as in eq. 10), and the 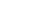 form to denote a specific class (e.g. 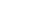). Similarly, the symbols 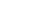, 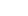 and 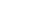 have the same meaning as 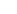, 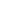 (eq. 11–12) and 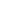 (eq. 14), respectively.

### Multiple Sequence Alignments

A set of ABC-B and a set of ABC-C protein sequences were collected from UniProt release 15.8 using HMMER3 [52]. In both the ABC-B and ABC-C family the “full transporter” is composed of two homologous “half transporters”, each of which contains a TMD and an NBD arranged as TMD-NBD (the “-” means that the domains are on the same subunit). But there are important differences between the two families. In in most ABC-B proteins the two halves constitute separate subunits (domain arrangement: TMD1-NBD1 TMD2-NBD2) while in all ABC-C proteins the halves are covalently linked (TMD1-NBD1-TMD2-NBD2). Moreover, in ABC-B proteins the two halves TMD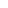-NBD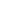 (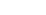) are in general identical or very similar to each other but in ABC-C proteins the halves have extremely diverged from each other. For these reasons, the ABC-B sequence set contained half transporters but the ABC-C set contained full transporters.

A separate multiple alignment (Dataset S1 and S2) was made from each set using MAFFT v6.717b [53] from which all gap-containing positions were removed while keeping the remaining positions aligned. The resulting ABC-B alignment contained 1585 sequences, the ABC-C alignment 553 sequences.

### Alignment Filtering

For each unfiltered alignment 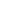 and filter type 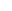, a sequence 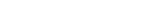 of filtered alignments was generated by removing 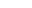 sequences, where 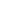 is the number of sequences in 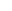. As mentioned above eq. 9, the type specifies the order of removal. The two types used in this work are called *phylogenetic filter* and *random filter* (Figure 4). As discussed before, the role of the phylogenetic filter employed in this work is to remove “sequence redundancies” from the alignment. In contrast, the random filter will be used to study how the performance of coevolution detectors depend on the number of aligned sequences.

In case of the random filter, the order of removal is given by a random permutation of sequences. The phylogenetic filter applies a deterministic permutation rule to the alignment 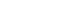 before the next sequence is removed and 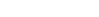 is generated. The rule is to consider the pair-wise evolutionary distance of all sequence pairs 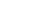, where 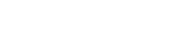 and 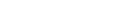. Next, the pair 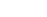 that has the shortest distance is found. Note that this is the most redundant pair according to the distance measure. Next, either 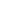 or 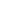 is swapped with 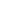 producing the new permutation. Removing the first sequence of the new permutation creates 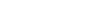 and completes the cycle. Thus 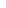 is decremented by one in each iteration of the cycle.

In terms of a phylogenetic tree, a single cycle is equivalent to finding the pair of tips connected by the shortest distance and stripping away one of these tips (with its terminal branch). As this cycle is repeated, filtering becomes “stronger”, the number of sequences decreases, and the minimal sequence-sequence distance 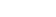 increases in the alignment (Figure 4B top graph).

To save computational time, only a subsequence of alignments 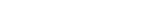 were analyzed with coevolution detectors. For 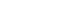, 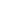 was chosen to be uniformly spaced (within rounding error) between 1 and 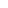, whereas 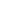 was set to 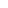 corresponding to the unfiltered alignment.

### Selected Coevolution Detectors

Three families of coevolution detectors were used in this study: CoMap [19], [38], mutual information (MI) [54] and CAPS [55]. The CoMap family is conceptually related to detectors in ref. [11], [14], [37]. This family contains detectors of the form CoMap-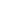-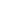, where 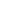 is either *correlation* or *compensation*; and 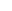 is either *simple*, *Grantham*, *polarity*, *volume* or *charge* [19]. Unlike other 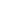
s, *simple* can be combined only with *correlation* but not with *compensation*. In this work CoMap-correlation-simple is referred to as CoMap. The mutual information family contains MI [54] and MIp [31]. The CAPS family, closely related to McBASC and other detectors [13], [16], consists of CAPS and CAPS-t, where “t” denotes time correction [55].

The selected detectors strikingly differ in whether, and how, they account for the non-independence of phylogenetically related sequences. CoMap accounts for this non-independence from “first principles”. This detector considers the set of branches 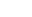 of a phylogenetic tree as a sample space on which, for each position 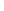, a random variable 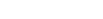 is defined, whose value is the expected number of substitutions that occurred along a given branch 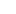. For each pair 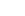 the statistic of CoMap is the correlation coefficient between 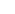 and 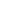. In contrast, MIp and CAPS-t uses empirical correction formulas, whereas MI and CAPS assumes statistical independence of sequences.

Another difference among detectors is related to the transition rates of the substitution process, which is intimately related to the physico-chemical similarities between amino acids. CoMap and CAPS allows realistic, heterogeneous rates by utilizing the empirical rate matrix of the WAG-F-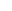 model. MI and MIp, however, assume the same rate for all types of transition.

Unfortunately not all detectors could be applied to all alignments. The time complexity of CAPS is 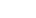, where 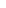 is the number of sequences in the alignment. This made alignments with 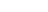 intractable for CAPS in the authors’ implementation [55]. Due to a segmentation fault, CoMap v1.3.0 [19] failed to run on alignments with roughly 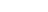 and with many variable positions. For these reasons only MI and MIp were applied to the large (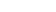) alignments of ABC-B sequences and a few variable positions, whose discretized substitution rate was typically 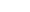, needed to be removed from the weakly filtered ABC-C alignments (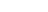). Consequently the size of certain rate classes, especially that of 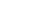, was smaller than others.

## Results

The procedures of the framework described above were carried out separately for the ABC-B and ABC-C protein family. The central goal of these procedures is the optimal detection of coevolving pairs of positions, given the sequence alignment data and the structural models representing each family, as well as the selected coevolution detectors. More specifically, the procedures search for the optimal parameter set 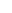 (eq. 5, 19), given a structural model and the set of contact pairs. As Figure 2A illustrates, 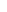 in general incorporates the parameters 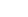, which determine the strength of phylogenetic alignment filtering (eq. 9), and the parameters 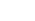, which control both the weights on substitution rate classes (eq. 11–13) and the weighted combination of detectors (eq. 15). Moreover, 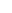 determines the set 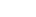 of optimally predicted coevolving pairs (Figure 2B) and thus set 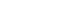 of pairs, which represents the coevolving subset of the known side chain contacts.

In what follows, the following questions are studied: To what extent do individual procedures improve the performance of coevolution detectors in the prediction of known contacts? What are the sources of improvement? Then, the pairs in 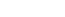 are further analyzed and presented in light of conformational changes.

### Extent and Sources of Improvement by Optimization Procedures

Figure 5 summarizes, for the ABC-C data set, contact prediction performance under 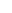 (magenta, optimal CoMap
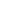
MIp) or under conditions lacking some or all of the optimization procedures. The receiver operating characteristic curves (Figure 5A) demonstrate that the relative performance under various conditions depends on the false positive rate 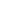, or reverse specificity. Consequently, the partial area 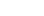 under these curves reports on the relative performance in a way that depends on the upper limit 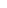 of integral of 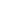 with respect to 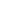 (eq. 18, Figure 5B). For most optimization procedures the relative improvement in performance was greater at high specificity (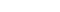, bottom bar graph) than at low specificity (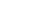, top bar graph). Importantly, 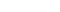 is more relevant to the predicted coevolving pairs (next section) because those represent the fraction 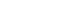 of all pairs (eq. 8), whose vast majority is not in contact (the structural model contained 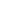 more distant pairs than contact pairs).

Figure 5 also demonstrates that all optimization procedures contributed to the improved performance under 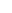. At 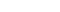, the greatest improvement was effected by the optimally weighted combination of CoMap and MIp, relative to using either of the two detectors alone. For computational efficiency (Text S1) the remaining 9 detectors were omitted from the weighted combination. Discarding these detectors may be justified by the result that they were clearly inferior to CoMap and MIp in performance (Figure 5 and Figure S5 and S6). At low 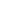 (Figure 5A) and at 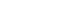 (Figure 5B) CoMap greatly outperformed even MIp. Despite this, the optimally weighted CoMap
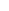
MIp performed markedly better than CoMap alone, which demonstrates the utility of weighted combination of detectors.

Figure 3 illustrates the principle of weighted combination of coevolution detector 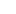 and 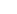, and presents performance for different relative weights. The figure takes as an example 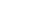 MIp and 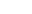 CoMap applied to substitution rate class 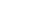 for the ABC-C family and demonstrates that equal weighting is not in general optimal. In this case, the equally weighted 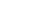 failed to induce any improvement in performance (circles in Figure 3B) in comparison with using 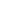 only. This result highlights the significance of (possibly unequal) detector weighting. As mentioned before, these effect were greater at low 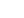 (compare Figure 3B to C).

To understand why phylogenetic filtering improved performance (Figure 5), it is useful to recall that this filter type was designed to remove the redundancies induced by closely related sequences, since these redundancies compromise the performance of all coevolution detectors. Figure 4 exemplifies the effects of alignment filtering for MIp; similar results were found for all other detectors (Figure S7 and S8). Comparing tree ***c*** to ***a*** in Figure 4A shows that strong phylogenetic filtering had a dual effect on the tree representing the alignment: (i) very short terminal branches (which indicate redundancies) disappeared but (ii) relatively few sequences remained in the alignment. The inverse relationship between effect (i) and (ii) was further established by applying the phylogenetic filter at gradually increasing strength (Figure 4B top).

Phylogenetic filtering had a dual effect also on performance (Figure 4B). Weak filtering (when the number remaining sequences 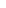 was between ca. 300 and 550) improved, whereas strong filtering (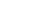) deteriorated performance. Both effects were more pronounced at 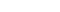 (bottom graph) than at 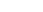 (middle graph).

The dual effect of the phylogenetic filter on both tree and performance suggested that the increase in performance was related to effect (i) on the tree, whereas the decrease in performance to effect (ii). This hypothesis was tested by applying the random filter, which was designed to dissect effect (ii) from (i). In line with this design, strong random filtering did not affect the distribution of the length of terminal branches (tree ***b***, Figure 4A). Performance (dashed lines in Figure 4B), however, deteriorated at increasing rate with respect to the strength of random filtering. This result, in agreement with the above hypothesis, suggests that the rate of performance deterioration by effect (ii) exceeds the rate of performance improvement by effect (i) at strong filtering. Therefore, optimizing phylogenetic filtering (by finding the maximum location 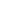) is equivalent to balancing these two rates (Figure 4B, bottom).

Partitioning position pairs (explained by Figure S2) into 10 substitution rate classes 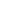 amplified the filtering-induced improvement in performance particularly in the case of CoMap (Figure 5). Consistently, 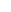 depended on 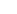 for all detectors, especially for CoMap (see empty circles marking 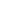 in Figure S8). This dependence is addressed by the combination of filtering and partitioning, which allows the conditioning of 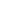 on 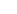 (eq. 14).

Another benefit of partitioning was related to the possibility of weighting classes. Optimal class weighting substantially improved the performance of CoMap, MIp and MI at 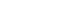 (Figure 5). The sources of this improvement were clarified by two further results. First, the distribution of the statistic of each detector clearly depended on 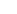 (Figure S3 and S4). Second, the conditional version of the performance measure 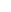 was calculated given each 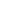 (Figure S7, S8 and in particular Figure S9). This uncovered the dependence of performance on substitution rate; the dependence was especially strong for CoMap. In light of these results, the advantage of class weighting is that it removes both types of dependence by conditioning threshold 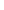 on 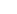 (eq. 14).

### Predicted Coevolving Pairs

When the fraction 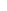 (eq. 8) of predicted position pairs was set to 0.001, 95 and 344 coevolving pairs were predicted for the ABC-B and ABC-C family, respectively. The roughly 4-fold difference between these numbers was due to neglecting the relatively small asymmetry between the two homologous halves of ABC-B proteins by creating an alignment from half ABC-B transporter sequences (Methods). Thus, for all pairs 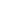, both position 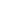 and 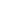 was restricted to the same half ABC-B transporter (this restriction was not used for ABC-C transporters, whose halves are greatly asymmetric).

The main focus of this study is not the entire set 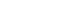 of predicted pairs but the subset 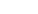, where 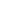 is the set of contact pairs observed in a representative structure. For the optimization procedures, 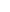 was calculated from the outward-facing Pgp and CFTR structures for the ABC-B and ABC-C family, respectively. 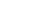 contained 41 pairs for the ABC-B and 95 pairs for the ABC-C family. For both families the positive predictive value 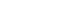 was an order of magnitude higher than the fraction 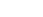 of contact pairs in the set 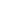 of all pairs. For example, for the ABC-C family 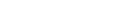 whereas 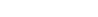. Consequently, the separation 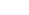 between predicted pairs 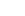 in 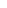-helices was distributed in a way that reflected 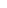-helical periodicity (Figure S10, Movie S1) [29], [36].

As a corollary of the unequal size of the 10 substitution rate classes 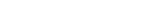 together with the weighting of these classes, the size of sets 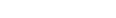 was also non-uniform. Most predicted pairs 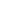 fell into class 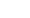 (Figure S1), whose definition (eq. 22) asserts either that the discretized substitution rate 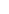 at position 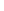 equals 3 and 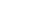 or that 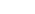 and 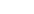. As expected, relatively variable positions (exhibiting 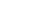 or 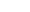) clustered mainly in the 12 transmembrane helices (TM1-TM12), whereas relatively conserved positions (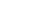 or 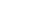) were typically located in the 4 intracellular loops (ICL1-ICL4) and the two NBDs, particularly at the central dimer interface (Figure 1B). The positions from which predicted pairs were composed tended to cluster also within the TM helices (Figure 1C). The latter finding, however, does not necessarily imply a natural tendency of coevolving pairs to reside in the TM helices. Rather, it can be seen as a consequence of the previous two results that link, via substitution rate, prediction sensitivity to structural localization.

For detailed exploration of the predicted coevolving pairs (Table 1, 2, 3, Dataset S5, S6), the set 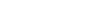 was considered, where 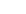 and 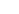 is the set of contact pairs in the outward and inward-facing conformation, respectively, of Pgp or CFTR. Thus all predicted pairs were included that were in contact in at least one of these two conformations. At the same time, 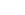, 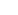 and
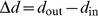
(23)were noted, where 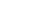 and 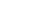 is the 3D distance separating pair 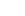 in the outward and inward-facing conformation, respectively. Therefore, 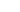 is the change of distance induced by the complete transition from the outward to the inward-facing conformation.

**Figure 6:**
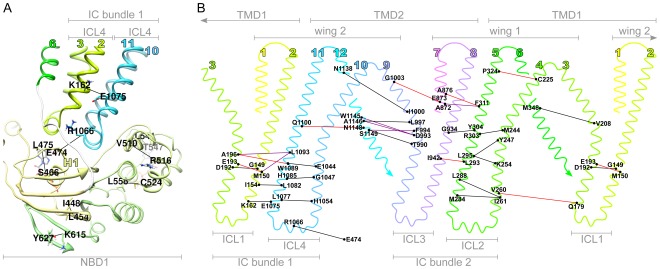
Coevolving position pairs in ABC-C proteins. (**A**) Labeled residue side chains connected by lines form subset 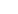 of predicted coevolving position pairs (eq. 24, Table 2) in NBD1, including (E474, R1066) that connects NBD1 to ICL4. Large colored numbers identify helices of the TMDs. Helix H1 of NBD1 is also labeled as in Figure 7. (**B**) The subset 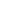 of predicted pairs (eq. 25, Table 3) are indicated in a topological map of the TMD dimer, in which 12 TM helices (large colored numbers), 2 wings and 4 intracellular loops (ICLs) are labeled. The map was obtained by cylindrical projection of the two polypeptide chains of the TMD dimer. Note that TM1-TM3 are shown twice. In both **A** and **B** the color of the lines connecting predicted pairs reports on the extent of distance change 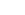 induced by the modeled outward 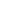 inward conformational transition (eq. 23). Black: 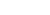; purple: 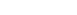; red: 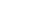.

For the pairs of the ABC-B family (Table 1) and for those in the NBDs of the ABC-C family (Table 2 and Figure 6A) the set of interest was further narrowed to
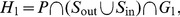
(24)where 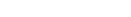, i.e the set of pairs fulfilling the condition that 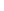 and 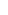 are separated by more than 4 positions in the sequence. This constraint removed “obvious” contact pairs, whose distance is constrained by primary rather than secondary to quaternary structure.

For the pairs of the TMDs of ABC-C proteins (Table 3, Figure 6B and Movie S2), a more restrictive condition was used to define the set 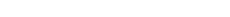. This means that the set
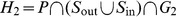
(25)contains those pairs 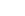 that were predicted to coevolve, for which 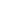 was observed to contact 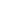 in at least one conformation, and for which 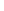 and 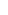 localized to distinct TM helices. In this case, the notion of a “TM helix” included the helices of the ICLs since those are contiguous extensions of the *sensu stricto* TM helices. Figure 1A and 6 show that each of the 4 ICLs contains two helical extensions and a single “coupling helix” [44], and that pairs of ICLs form compact structural units that predominantly interact with a single NBD: 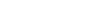 with NBD1 (Figure 6A) and 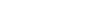 with NBD2. These units of 4 parallel helices are hereby termed *intracellular bundle* 1 and 2 consistently with the interacting NBD.

**Table 1:** Coevolving Position Pairs in ABC-B transporters.

position 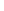				position 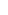				3D distance (Å)		
Pgp-N	Pgp-C	region	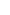	Pgp-N	Pgp-C	region	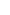	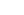	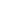	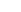
TMDs										
A58	A718	TM1	2	Q195	Q838	TM3	2	5.2	11.7	–6.4
I59	I719	TM1	3	G124	I765	TM2	3	15.5	5.4	10.1
F151	V792	ICL1	2	I369	I1012	TM6 ext.	1	7.1	11.1	–3.9
Q158	Q799	ICL1	0	N371	K1014	TMD1-NBD1	2	6.3	13.8	–7.5
S228	A871	TM4	2	A301	F944	TM5	3	5.5	9.3	–3.8
L236	L879	TM4	2	T294	I937	TM5	3	5.7	9.6	–3.9
T240	A883	ICL2	2	A361	S1004	TM6 ext.	1	7.2	27.1	–19.8
D241	L884	ICL2	3	Y363	A1006	TM6 ext.	3	6.0	30.2	–24.1
NBDs										
E393	T1036	S1	3	K416	E1059	S2	3	5.4	22.2	–16.8
R395	G1038	S1	3	M450	K1093	S4	3	5.1	21.3	–16.1
N396	E1039	S1	2	G412	G1055	S2	2	5.8	22.5	–16.7
H398	V1041	S1	3	G412	G1055	S2	2	5.3	26.0	–20.6
H398	V1041	S1	3	E448	A1091	S4	3	3.6	22.6	–19.0
S400	N1043	S1–S2 loop	3	T447	L1090	H1–S4 loop	3	4.7	21.5	–16.8
K411	Q1054	S2	3	V605	R1250	S10	3	5.5	7.5	–1.9
L415	L1058	S2	2	A599	V1244	S9	2	6.6	27.8	–21.1
Q421	Q1064	S2–S3 loop	2	V597	L1242	S9	3	6.7	5.6	1.0
V423	L1066	S3	1	V597	L1242	S9	3	5.1	3.8	1.3
V437	V1080	H1	2	L553	L1198	S7	1	5.3	15.0	–9.6
M450	K1093	S4	3	D457	E1100	S5	2	5.4	8.7	–3.2
A485	A1128	H3	3	D521	S1166	X-loop	2	6.3	17.3	–11.0
N508	N1153	H4–H4b loop	1	V568	V1213	H6	3	4.3	12.1	–7.7
V597	L1242	S9	3	K609	H1254	S10	2	6.4	19.2	–12.8

These position pairs 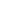 form subset 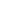 of the predicted coevolving pairs in the ABC-B family. By definition (eq. 24), 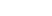 means that 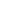 and 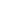 are in contact in either the outward or inward-facing conformation and are separated by more than four positions in the sequence. Because the ABC-B alignment contained only half transporter sequences, no pairs were predicted between the N and the C terminal halves. *Pgp-N* and *Pgp-C*: residues and positions are given for both the N and the C terminal half of human Pgp (UniProt ID: MDR1_HUMAN), respectively. The Pgp-N or Pgp-C position numbers can readily be converted to position numbers of other ABC-B half transporters using the mappings given by Dataset S3. 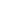 and 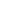: discretized substitution rate (eq. 20) at position 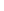 and 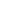, respectively; *3D distance*: between position 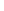 and 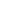; 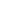 and 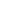: distance obtained from structures representing the outward [47] and inward-facing [46] conformation, respectively; 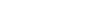 (eq. 23). A more extensive presentation of predicted pairs is available in Dataset S5.

**Table 2:** Coevolving Position Pairs in the NBDs of ABC-C transporters.

position 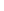				position 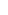				3D distance (Å)		
CFTR	region	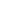	ref.	CFTR	region	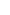	ref.	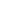	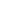	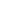
I448	S2	3		L454	S3	3		5.1	5.1	0.0
S466	H1	1		L475	H1–S4 loop	2		7.8	7.8	0.0
V510	H3	3	[67], [69]	R516	H4	3		7.3	7.2	0.1
C524	H4	2	[66]	L558	H5	1		4.8	4.9	−0.1
L541	X-loop	1		T547	C-loop	2		5.9	5.9	0.0
K615	H7–S9 loop	4		Y627	S10	3		6.8	6.8	−0.1
L1242	S3	2		I1398	S8	2		6.1	6.0	0.0
E1321	H4	3		A1391	H6	3		7.7	8.0	−0.2
K1389	H6	2		E1409	H7	2		6.4	6.2	0.1
L1399	S8	1		C1410	H7–S9 loop	2		5.8	5.7	0.1
E474	H1–S4 loop	2		R1066	coupl. H (ICL4)	1	[71]–[73]	7.5	9.3	−1.8

The table list those pairs 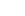 of the set 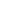 (eq. 24), for which either 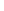, 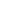 or both are located in an NBD of ABC-C proteins. For all of these pairs, except for (E474, R1066), both 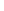 and 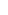 was found in the same NBD. 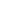-helices (H) and 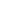-strands (S) are numbered according to ref. [74]. *CFTR*: residues and positions are given for human CFTR (UniProt ID: CFTR_HUMAN). These position numbers can readily be converted to position numbers of other ABC-C transporters using the mappings given by Dataset S4. Other columns have analogous meaning to those in Table 1 with the distinction that for this family the outward and inward-facing conformation correspond to the models described by ref. [45] and [48], respectively. A more extensive presentation of predicted pairs is available in Dataset S6.

**Table 3:** Coevolving Position Pairs in the TMDs of ABC-C transporters.

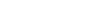	position 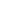				position 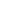				3D distance (Å)		
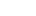 or 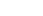	CFTR	ICL 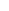	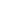	ref.	CFTR	ICL 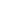	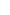	ref.	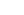	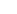	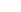
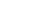 or 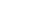	E873		3		G1003		4		14.8	5.8	9.0
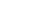 or 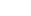	A872		3		F311		3		9.5	4.8	4.7
	A876		4		F311		3		12.7	5.4	7.3
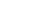 or 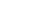	G149	1	3	[68]	D192		3		5.3	6.4	−1.1
	M150	1	3		E193		4		13.3	6.0	7.3
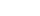 or 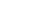	M150	1	3		L1093		4		7.4	12.7	−5.4
	I154	1	3		L1082	4	3		5.7	3.7	2.0
	K162	1	3		E1075	4	4		5.7	6.7	−1.0
	G934		3		Y304		3		7.3	9.5	−2.3
	I942		3		L293	2	3		12.4	6.4	6.0
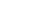 or 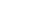	Q179	1	3		V260	2	3		5.7	16.1	−10.4
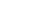 or 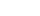	V208		3		M348		4		7.6	7.4	0.2
	T990		4		S1149		3		7.0	6.8	0.2
	D993		4	[59]	W1145		3		8.1	5.0	3.1
	D993		4	[59]	A1146		3		10.6	6.2	4.4
	F994		3		S1149		3		5.1	8.3	−3.2
	L997		4		A1146		3		5.7	7.7	−2.0
	I1000		4		N1138		3		5.6	5.4	0.2
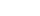 or 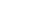	A196		4		W1089	4	4		13.5	7.0	6.5
	A196		4		L1093		4		11.4	7.7	3.8
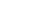 or 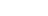	C225		3		P324		3		4.9	12.7	−7.7
	M244		3		R303		3		6.9	8.0	−1.2
	Y247		4		L295	2	4		7.1	7.0	0.1
	K254	2	4		L295	2	4		5.7	7.5	−1.9
	I261	2	3		M284	2	3		6.9	9.7	−2.8
	I261	2	3		L288	2	3		5.3	8.4	−3.1
	E1044	4	2		W1089	4	4		5.5	5.9	−0.3
	G1047	4	3		H1085	4	2		4.7	3.7	1.0
	H1054	4	2		L1077	4	3		7.2	8.6	−1.3
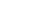 or 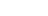	Q1100		3		N1148		2	[68]	7.9	16.1	−8.2

These position pairs 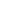 form subset 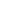 of the predicted coevolving pairs in the TMDs of the ABC-C family. By definition (eq. 25), 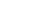 implies that 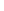 and 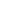 are in contact in either the outward or inward-facing conformation and are located in separate TM helices. Here the notion of a “TM helix” includes the helices of the ICLs. The left column contains the indices 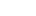 of each TM helix pair (TM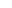,TM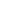) together with the indices 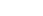 of the homologous helix pair. *ICL* 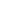: this column contains the index 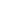 whenever position 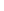 falls into ICL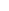; *ICL* 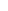 has analogous meaning for position 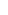. For the description of all other columns see Table 1 and 2. A more extensive presentation of predicted pairs is available in Dataset S6.

### Pairs Involved in Conformational Changes

**Figure 7:**
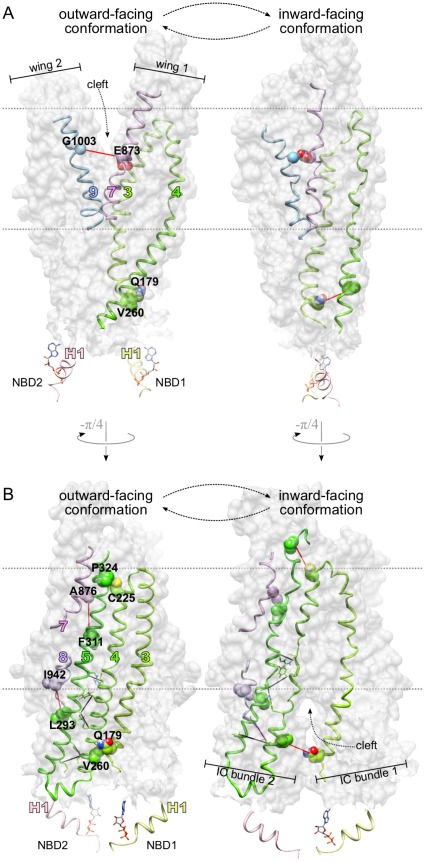
Position pairs evolved to regulate conformational transitions. (**A-B**) The entire TMD dimer is shown in surface representation, and selected TM helices (identified by colored numbers) are displayed as ribbons. For each NBD, only helix H1 (cf. Figure 6A) is shown, as well as the bound ATP, if present. The outward-facing model conformation is characterized by a cleft between wing 1 and 2 (**A**, left) while the inward-facing model conformation reveals a cleft between intracellular bundle 1 and 2 (**B**, right). All labeled position pairs (connected by red lines and represented as spheres) were predicted as coevolving, and represent structural contact in only one of the two conformations, suggesting that these pairs evolved to regulate conformational transitions. Unlabeled pairs (black connecting lines, stick representation) are expected to remain in contact in both principal conformations, implying that they evolved to enhance structural rigidity. (**A**) (E873, G1003) and (Q179, V260) appear to regulate the opening and closing of the cleft between the wings and that between the IC bundles, respectively, during the conformational change. (**B**) (C225, P324), (F311, A876) and (L293, I942) might regulate the relative translation of TM4, TM5, TM7 and TM8 along the helical axes.

Comparison of the CFTR structural models in the outward and inward-facing conformation (Movie S2) revealed possible conformational transitions [48], [56]. The most striking change during the inferred outward 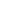 inward transition was the dissociation of the tight dimer of NBDs, the closure of the outward-facing cleft delineated by the wings (Figure 7A) and the opening of the inward-facing cleft between the intracellular bundles (Figure 7B). While the NBDs and the lower (i.e. proximal to the NBDs) parts of the IC bundles moved as essentially rigid bodies, the upper parts of IC bundles and especially the wings appeared flexible. A prominent component of that flexibility was the translation of some TM helices along their axes relative to other helices.

These inferred movements during the outward 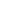 inward transition were quantified by the distance change 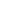 (eq. 23), whose extent 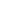 is indicated by the color of the line connecting each pair in Figure 6, 7 and Movie S1, S2, S3. In Table 2, 3, Figure 6, 7 and in the main text below residues and positions are given for human CFTR (UniProt ID: CFTR_HUMAN), whereas homologous positions for 599 other ABC-C proteins can be obtained from Dataset S4. (E873, G1003) and (Q179, V260) stood out among the pairs in 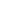 (and in fact also in 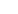), for which 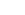 was relatively large (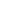
Å, red lines). The uniqueness of these two pairs was established by the fact that they contributed to the structural contacts between the closed wings and IC bundles, respectively, but were separated by the cleft between the wings/bundles in the opposite conformation (Figure 7A-B, Movie S3).

For the rest of the red pairs 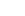 in 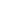, position 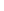 resided in the same IC bundle or wing as 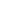 (Figure 6B). These included (L293, I942) in IC bundle 2, as well as (C225, P324) and (F311, A876) in wing 1. As Figure 7B and Movie 24 illustrate, the separation of these conformation-specific contact pairs was due to the inferred bending and translation of TM helix 5 with respect to TM7 and TM8. TM4 and TM5 was unusual in that they exhibited marked translation relative to each other at their extracellular ends, containing (C225, P324), whereas the same helix pair appeared relatively rigid in ICL2 (see the 4 unlabeled black and purple pairs in Figure 7B). In this regard, ICL4, formed by TM10, TM11 and a coupling helix directly interacting with NBD1, was similar to ICL2 (Figure 6B). Notably, the coupling helix of ICL4 contains R1066, which together with E474 formed the only pair in 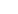 that links an NBD to any other domain; 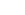 was relatively small for this pair too.

## Discussion

The new framework employed in this study is *integrative* in at least two ways. In one sense, it allows joint analysis of sequence and structural data for some protein family. In another sense, the framework integrates over several detectors by combining them in a weighted manner. In both senses, the present work surpasses previous studies, which analyzed sequence and structural data separately and used either a single detector [11], [18]–[25] or a combined detector with equal weights [30].

How does joint analysis of sequence and structure aid the prediction of coevolving position pairs? A long-standing challenge to accurate prediction of coevolving positions has been the lack of trusted datasets on coevolution, which could help optimize the sequence-based coevolution detectors. The new framework attempts to overcome this obstacle by making use of a solved structure and defining the objective function of the optimization in terms of the prediction of known contact pairs (eq. 5, 19). The justification of this approach certainly requires some assumptions as already discussed (eq. 1–6), but these assumptions are rather weak. In particular, it is not assumed that the set of side chain contacts contain pairs that are equally tightly coupled in terms of coevolution. On the contrary: the ultimate goal of the present approach is to distinguish contact pairs that coevolve tightly from contact pairs that evolve quasi-independently. Note, however, that the new framework is inapplicable to *de novo* structure prediction problems as it relies on an existing contact map.

In its present form, the new framework takes a single input structure, representing only one conformation and only one member of the analyzed protein family. How would an alternative input structure (from the same family) influence the predictions? Although the present work does not address this question in depth, preliminary analysis indicates that switching to a different input structure affects roughly 10 to 35% of the predicted pairs depending on how different the alternative structure is relative to the original one (Figure S11). This raises the question: when multiple structures or structural models are available within a protein family, which one should be selected as structural input? Intuitively, high resolution X-ray structures are expected to be more useful inputs than lower resolution X-ray structures or homology models, and this difference might be manifested in the performance of contact prediction. Comparing a few X-ray structures and homology models in the ABC-B (Figure S12) and ABC-C (Figure S13) family indicates some differences in performance. Remarkably, performance with the 3.8 Å Pgp X-ray structure (3G5U) [46]) was lower than that with the 3.0 Å Sav1866 X-ray structure (2HYD) [44] or with the Pgp homology models [47], whose TMDs were based on the same Sav1866 structure. It remains to be determined how structural heterogeneity of homologs, as well as conformational heterogeneity within each homolog, can be accounted for to improve the prediction of coevolving residues.

Recent studies [8], [9], [19]–[21], [40], [42], [57] presented sophisticated approaches for the prediction of higher order coevolving *networks* instead of merely coevolving *pairs*. Some of these reports [8], [9], [40], [42] demonstrated that accounting for higher order interactions vastly improved contact prediction performance. Although the present framework ignores higher order networks, this may not undermine its power substantially because it uses contact prediction only to optimize the parameters that control coevolution detectors. It remains an open question to what extent these parameters are influenced by ignoring networks. Without doubt, the ability to infer whole networks of coevolving positions would be beneficial for the clarification of biophysical mechanisms and even for rational design of mutants, although experimental testing of ternary or higher order interactions is usually impractical (but see ref. [1]).

The new framework is quite general as it can in principle incorporate optimization procedures in addition to the three procedures used in this study: alignment filtering, class weighting and detector weighting (Figure 2A). While class and detector weighting are novel procedures, phylogenetic filtering has already been employed by the majority of published analyses of residue coevolution but with crucial differences to the current work. In all previous analyses, except ref. [22], the strength of filtering was determined by “rules of thumb”, which may have lead to under or overfiltering and thus to a decline in performance, relative to even the unfiltered alignment. Moreover, it was previously ignored that the optimal filtering strength may depend on substitution rate and the selected coevolution detector, as demonstrated here (Figure S8).

Random filtering in the present work (Figure 4 and S8) revealed how performance scales with the number of sequences in the alignment [22]. The scaling itself depended both on substitution rate and the selected coevolution detector. CoMap showed the highest rate of improvement with increasing number of sequences, at least at those rates that were associated with the highest performance (Figure S8). This result suggests that CoMap can make use of the growth of sequence databases more efficiently than the other selected detectors. The same result also indicates that relatively parameter-rich, “tree-aware” detectors (like CoMap [19], [38] and those in ref. [11], [20], [36], [37]) depend more strongly on data quantity, and therefore their advantage over “tree-ignorant” detectors might have been overlooked previously [29].

Even though patterns of protein evolution may change over time, modeling time-variable patterns at the sequence level is already challenging when it is assumed that positions do not coevolve (see ref. [58] for insights). Therefore, until now, all coevolution detectors, including those in the present work, have been designed with the assumption that (co)evolutionary patterns are constant over time (i.e. persistent).

The assumption of time-invariance hinders the physico-chemical interpretation of certain pairs predicted to coevolve, while allowing time-variable patterns provides an explanation for these pairs, namely that they became coevolving from independent (or vice versa) in some lineages over time. A prime example is the pair in ABC-C proteins that corresponds to (E873, G1003) in human CFTR (Table 3 and Figure 7A), which may have become independent from coevolving as CFTR diverged away from other ABC-C proteins. Conversely, (R352, D993) was experimentally shown [59] to form a functionally important salt bridge in CFTR and yet the present analysis predicted D993 to coevolve with W1145 and A1146 rather than R352 (Table 2). But this contradiction is solved by the prediction [59] that D993 is involved in the functional divergence of CFTRs from other ABC-C proteins. For some predicted pairs, however, physico-chemical interpretation is straight-forward; e.g. (E474, R1066) in human CFTR may form a high-energy salt bridge in the solvent-inaccessible, hydrophobic interface between NBD1 and the coupling helices of two intracellular loops (Figure 6A).

Although coevolution detectors assume time-invariance, the present work did account for those changes in evolutionary patterns that occurred during long divergence processes following ancient gene duplications. As standard phylogenetic analysis suggests (Figure S14), one such duplication is the divergence of the ABC-B and ABC-C families from each other, which was followed by the divergence of the N and C terminal half transporters within the ABC-C family. These early events were taken here into account by creating separate alignment for (i.) ABC-B half transporters and (ii.) the N as well as (iii.) the C terminal ABC-C half transporters. (Note that the sequences in (ii.) and (iii.) are not separate in the sense that they form a single, “concatenated” alignment of full transporters). This approach is equivalent to ignoring the distant homology among the three clades of half transporters and has the disadvantage that those pairs cannot be identified that have persistently coevolved throughout the entire shared history of the ABC-B and ABC-C family. A related drawback is that it cannot be determined whether a predicted pair in one group of half transporters corresponds to some pair in another group, and so it cannot be studied how residue coevolution relates to the functional asymmetry between ABC-C half transporters.

All coevolution detectors use certain assumptions on the relative rates of substitution between different amino acids. The present work used CoMap with the WAG matrix [50], which derives substitution rates empirically from a large and diverse set of globular protein families. It remains to be determined to what extent this affects predictions of coevolving positions in the transmembrane domains of ABC transporters and other membrane proteins, and how the predictions would be improved by using empirical transmembrane-specific substitution matrices. The effect might be small if one considers that empirical matrices are much more similar to each other than to a “flat” matrix corresponding to unrealistic, uniform substitution rates, which is assumed by some detectors like MI.

Structural dynamics received little attention in previous coevolution analyses [8], [23], [37], [60]. Together with a recent study [61], this report presents one of the first quantitative and systematic treatment of this question. Two classes of coevolving pairs were predicted that are distinguished by the extent 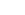 of the 3D distance change induced by the transition between opposite-facing conformations of ABC transporters. A simple functional interpretation is that the pairs with small 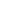 are evolutionarily conserved interactions that stabilize relatively rigid structural elements, in particular the NBDs and the intracellular bundles. In contrast, the positions of pairs with large 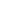 appear to have coevolved with each other to stabilize selectively one (set of) conformation(s) and thus directly regulate the structural dynamics of substrate transport.

The prevalent mechanistic model of ABC transporters [3]–[5] emphasizes a rigid-body movement of the TMDs, which is characterized by the alternate opening and closing of the cleft between the two wings and that between the two intracellular bundles, respectively. However, only two of the predicted pairs appear to regulate the opening and closing of these clefts directly (Figure 7A). The rest of pairs with large 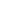 (Figure 7B) were inferred to regulate relative movements of helices within the same wing or intracellular bundle. This result points toward a more refined view of conformational changes, in which TM helices bend and translate along their axes, especially in the wings, which appear to be relatively flexible.

The predicted coevolving positions in the ABC-C protein family are given here (Table 2 and 3) in terms of the sequence of human CFTR, which functions as an ion channel as opposed to all non-CFTR ABC-C proteins, which are active transporters. While this does not affect the set of predicted pairs (which can be expressed in terms of any ABC-C protein sequence using the mappings given by Dataset S4), the functional difference must be borne in mind at the mechanistic interpretation of the predictions. Since CFTR diverged away from the canonical transporter function of the family [59], it is reasonable to speculate that some fraction of coevolving pairs became uncoupled in the CFTR lineage during the divergence. Exactly what fraction of coevolving pairs has been affected depends on the extent of structural changes that conferred CFTR with its novel function, which awaits to be clarified by future structural work on CFTR. Supported by the strict coupling between ATP hydrolysis and channel gating [62], it has been hypothesized that the gating of CFTR is essentially the same as the alternating-access mechanism of an ABC-C transporter, whose internal gate has been broken by evolution [59], [63]. Note that the gating mechanism itself is unaffected by the regulatory (R) domain [64], another unique feature of CFTR in the ABC-C family. If the “broken gate hypothesis” holds, the extent of the function-changing structural alterations may be quite subtle, as found in the CLC channel/transporter family [65].

Recent work [26]–[28] showed that the combination of coevolution analysis with double mutant experiments can be a powerful tool to clarify mechanistic details of ABC proteins, although these studies focused only on a few predicted pairs in the NBDs, and in one case [26] the predicted coevolutionary coupling was not strongly supported by experimentally measured biophysical coupling. The current work offers a more complete and systematic coevolution analysis on ABC proteins. Several pairs presented here are formed by positions, at least one which was previously reported to be important for normal structure and function (see references in Table 2, 3), which hints at the practical value of the predictions. Moreover, these positions were implicated in cystic fibrosis-related folding defects of NBD1 [66], in the correction of these defects [67]–[69] and, as mentioned above, in CFTR channel gating [59].

This work introduces a new, integrative framework for accurate prediction of coevolving position pairs, and applies it to the ABC-B and ABC-C protein families. Each predicted pair can be interpreted as a side chain interaction that regulates some static or dynamic property of protein structure. Future experiments using site-directed mutations at these position pairs may illuminate mechanistic details that are conserved and salient features of these protein families.

## Supporting Information

Figure S1
**Optimization with a differential evolution algorithm.** The figure shows independent runs, under various conditions defined by the control parameters of the algorithm, of the search algorithm for the optimal set 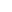 of thresholds used by some coevolution detector. 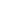 is defined as 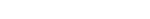, where each 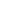 is the coevolution threshold (eq. 11) corresponding to substitution rate class 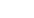 (eq. 22). Note that 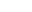 and so 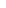 is a subset of parameters *for coevolution prediction* and is therefore not to be confused with the set of *control* parameters. The overall conclusion from this figure is that the solution 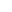 identified by this heuristic algorithm is a good approximation of the global optimum. (**A**) The algorithm was run independently 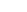 with the same control parameters as those used for the predicted pairs presented in Table 1, 2, 3. Each run was terminated at the 1000th generation (i.e. iteration). Top graph: improvement of population fitness (defined in Algorithm 1 of Text S1) in all 12 runs. The rate of improvement declined after a few hundred generations suggesting that 1000 generations are sufficient. Bottom: the evolution of 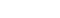 is shown for one of the 12 runs (identified by black color in top graph). 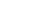 is the set of predicted coevolving pairs in class 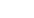 and so this graph further supports the previous conclusion from the top graph. (**B**) The approximate 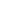 appears to lie close to the true optimum since 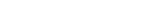, where 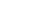 is a random sample of size 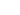. (**C**) 1st generation (left): each of the 12 independent run was initialized from a distinct, randomly chosen, position of the parameter space. 1000th generation (right): all runs converge to nearly the same 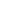, indicated by 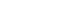. This suggests that the solution is robust against the randomness inherent to the initialization of the algorithm. (**D**) The solution appeared to be robust against also the control parameters of the algorithm.(TIF)

Figure S2
**Partitioning the set of position pairs into substitution rate classes.** (**A**) Substitution rate at all 880 single positions (gray horizontal symbols) present in the ABC-C protein sequence alignment. The figure demonstrates that the substitution rate 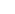 varies greatly with the position index 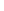 (here the expected 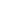 is shown, which was obtained by the empirical Bayes approach [51], and normalized to 1 over all 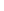). As expected (eq. 20–21), the estimated discretized substitution rate 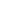 (eq. 21) correlates with 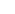. (**B**) Classes 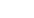 of pairs can be defined (eq. 22) using 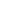 and 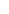 for each of the 386760 position pairs 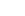. Since 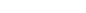, there are 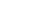 classes and therefore, using a scalar index 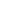, the partitioning results in the collection 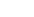 of classes 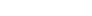.(EPS)

Figure S3
**Dependence of coevolution statistics on substitution rate.** Distribution of the standardized statistic for 4 distinct coevolution detectors (CoMap, MI, MIp and CAPS). Red line: distribution over all pairs of positions. Each blue line corresponds to the distribution over a specific rate class 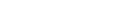.(EPS)

Figure S4
**Dependence of coevolution statistics on substitution rate: tail of distribution.** The graphs from Figure S3 have been expanded to illustrate the effect of substitution rate on statistical errors. Taking MIp as an example, point 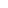 marks the upper 1st percentile of the red distribution, calculated from all pairs. Setting the threshold 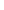 to the black vertical line for all pairs is equivalent to expecting the false positive rate 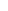 at 0.01. But since the distribution of the coevolution statistic varies substantially with substitution rate (see the dispersion of blue lines here and in Figure S3), 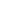 also varies at a fixed threshold. At the vertical black line, for example, 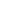 ranges between point 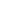 and 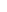. Therefore the prediction is biased toward certain rate classes, such as the one identified by point 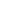. This bias is addressed by setting a distinct threshold 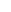 for each class 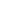 (eq. 11).(EPS)

Figure S5
**Performance of variants of CoMap.** The figure demonstrates that CoMap (a shorthand for CoMap-correlation-simple) outperformed other CoMap variants. These variants differ from each other in the type of coevolution statistic (correlation or compensation) and the physical quantity of the amino acid side chain that is used for the weighting of substitution vectors during the evaluation of the statistic [19]. This particular set of results corresponds to rate class 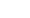 but similar findings were obtained for all other classes.(EPS)

Figure S6
**Performance of variants of CAPS.** The graph presents findings from a previous alignment of ABC-C protein sequences, to which a phylogenetic filter was applied. This phylogenetic filter is essentially the same as the one described in the main text and illustrated by Figure 4 except that in this case the sequence-sequence distance was expressed as (reverse) percent identity instead of the maximum likelihood estimate of the number of substitutions per position (Figure 4B top graph). In the filtered alignment the closest sequence pair had 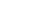 identity and the time correction had essentially no effect on performance. Then a single sequence (which was previously removed by the filter) was reintroduced to the alignment. This sequence was 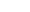 identical to some other sequence in the alignment. The bottom bar shows that time correction worsened performance to the level of a random detector. In summary, this figure demonstrates that the time correction of CAPS had either no advantage or it had an adverse effect on performance.(EPS)

Figure S7
**Random filter: performance as a function of several variables.** This and the next figure explores the dependence of 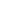 on three “independent variables”: the number of remaining sequences (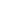 axes), the substitution rate (individual graphs labeled with a particular rate class 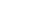) and the choice of coevolution detector (color of lines). Each solid line shows how performance scales with the number of sequences in the alignment when the distribution of sequence-sequence distance is *independent* from this number. These results correspond to the ABC-C family.(EPS)

Figure S8
**Phylogenetic filter: performance as a function of several variables.** This figure is analogous to Figure S7. Each solid line shows how performance scales with the number of sequences in the alignment when the distribution of sequence-sequence distance also *depends* on this number (cf. top graph in Figure 4B). The circles indicate the optimal number 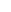 of remaining sequences (cf. bottom graph in Figure 4B).(EPS)

Figure S9
**Dependence of performance on substitution rate.** This bubble plot shows performance, gaged by 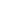, as the area of the circles. Performance was conditioned not only on the choice of coevolution detector (individual graphs) but also on substitution rate class (position of the circles within each graph). In principle, conditioning on rate class removes the dependence of the statistic on substitution rate (Figure S3, S4) and so dissects out the dependence of performance. Note that relative performance is displayed and that the scale at the right bottom corner depicts the area of circles that is equivalent to 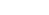 and 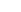 better performance than that of a random detector. The black (empty) circles represent performance at optimal phylogenetic filtering. Inside these circles gray (filled) disks represent performance without any filtering. These results correspond to the ABC-C family and should be compared to Figure S8.(EPS)

Figure S10
**Periodicity of** 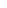 **-helices.** The histograms show the distribution of the separation 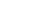 in sequence for pairs 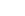 in the set 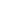 of predicted coevolving pairs (**A** and **C**) or in the set 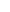 of contact pairs (**B** and **D**). On the left the subset 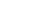 (**A**) and 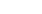 (**B**) is shown where 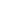 is the set of pairs 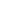 for which both 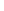 and 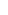 are located in the same helix. On the right **C** and **D** shows analogous subsets for loops instead of helices. Comparing the shapes of distributions it is clear that **A** is similar to **B**, and **C** to **D**; the resemblance is due to the high fraction of contact pairs in 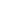. Comparing **A** to **C**, and **B** to **D** reveals a peak at 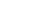 or 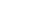 and a valley at 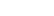 in **A** and **B** but not in **C** and **D**. The peak corresponds to one helical turn, whereas the valley half a turn.(EPS)

Figure S11
**Effect of the input structure on the set of predicted pairs.** The figure shows how the set of predicted coevolving pairs depends on the input structure. Consistency of an input structure 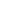 with the reference structure 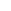 is defined as 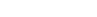, where 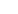 and 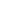 is the set of predicted pairs using 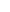 or 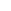 as structural input, respectively. When the input and reference structure is the same (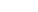), consistency is 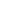 (points at the upper left corner). But when the input and reference structures differ from each other, consistency decreases to a value that depends on the RMSD difference between the structures. Even in the “worst case” (Pgp: 3F5U) consistency is about 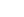, meaning that on average two out of three pairs predicted with the reference structure are also predicted with the alternative input structure.(EPS)

Figure S12 **Effect of the input structure on performance in the ABC-B family.** This figure compares different input structures with the same detector (MIp) as opposed to Figure S8, which compares different detectors with the same input structure. Sav1866: 2HYD [44] and Pgp: closed [47] represent the outward facing conformation while Pgp: semiopen [47], Pgp: open [47] and Pgp: 3G5U [46] correspond to various inward facing conformations.
(EPS)

Figure S13 **Effect of the input structure on performance in the ABC-C family.** This figure is analogous to Figure S12 with ABC-C instead of ABC-B family. The input structural models were taken from ref. [45] and [48].
(EPS)

Figure S14 **Divergence of half transporters during the shared history of the ABC-B and ABC-C family.** This phylogenetic tree, created by the neighbor joining algorithm, shows the evolution of ABC-B and ABC-C half transporters. Although the tree is unrooted, a plausible scenario is that the common ancestor of the ABC-B and ABC-C half transporter family resides on the red branch. Following an ancient gene duplication, the two families started to diverge from each other. A subsequent duplication and gene fusion, where the red branch meets the blue branches, lead to the divergence of N and C terminal half transporters within the ABC-C family. These events created three distantly related clades of half transporters (grey shade). To avoid complications arising from functional divergence, residue coevolution was analyzed separately for each clade in the present work.
(EPS)

Text S1
**Heuristic search strategy for the optimal parameter set** 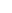 **.** The text describes a stepwise strategy for obtaining an approximate 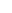. The differential evolution search algorithm of the last step is presented as pseudocode.(PDF)

Movie S1
**Predicted pairs** 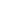 **with separation** 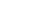 **in sequence.** The ribbon represents the polypeptide chain of CFTR in outward-facing conformation, and its colors match with those in Figure 6, 7 and Movie S2, S3. Residues in stick representation, connected by straight black lines, are position pairs predicted to coevolve in the ABC-C family and separated by 4 or fewer positions in sequence. For many pairs the separation occurs at one turn in an 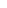-helix (Figure S10A). ATP molecules are shown in sphere representation.(MOV)

Movie S2
**Predicted pairs** 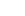 **with separation** 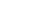 **in sequence.** The straight lines connect pairs contained in subset 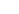 (eq. 25). As in Figure 6, black, purple and red connecting lines indicate the extent 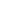 to which the 3D distance between 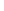 and 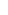 changes during conformational transition. The transition is modeled here by linear interpolation (morph) between the inward and outward-facing conformations.(MOV)

Movie S3 **Opening and closing of the wings and intracellular bundles of the TMDs.** As Movie S2, but showing only the same two pairs (sphere representation) as Figure 7A. Note that the cleft between the wings opens as that between the intracellular bundles closes and *vice versa*.
(MOV)

Dataset S1 **The ABC-B alignment.** Note that all gap-containing columns have been removed.
(FA) Click here for additional data file.

Dataset S2 **The ABC-C alignment.** Note that this alignment contains full transporters.
(FA) Click here for additional data file.

Dataset S3
**Positions of the ABC-B alignment.** This text file is a modified version of the unfiltered alignment (Dataset S1) of ABC-C protein sequences. The modification was to substitute, for each position and sequence, the one-letter amino acid code with the position number (position numbers are separated by commas). Therefore, this modification allows one to “translate” pairs of coevolving residue numbers in terms of Pgp (Table 1) to that in terms of any other ABC-B protein that is represented in this dataset. This is done simply by mapping residue numbers of Pgp-N (i.e. MDR1_HUMAN_N) to alignment column numbers and then column numbers to residue numbers of any protein 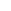 of interest; symbolically: position(MDR1_HUMAN_N) 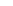 column 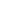
position(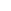). Sequence names are given as UniProt IDs, such as MDR1_HUMAN (Pgp). “Full transporters” are represented by both of their halves: the N and the C terminal one. To distinguish between these two, the ID of the N terminal half was extended with an “_N” appendix, like MDR1_HUMAN_N. Gaps had been previously removed from this alignment, which rendered several sequences to be identical to each other, even though the corresponding full sequences were not identical. Each set of “quasi-identical” sequences gave rise to an equivalence class. In the present text file, all sequences are listed within each equivalence class. For the analysis, however, only one sequence was considered in each class while the rest was removed.(TXT)

Dataset S4 **Positions of the ABC-C alignment.** This is a modified version of the ABC-C alignment (Dataset S2). See Dataset 28 for further explanation.
(TXT)

Dataset S5
**List of all predicted coevolving pairs in the ABC-B family.** Each Excel sheet lists the predicted coevolving pairs (including those not in structural contact) for a given fraction 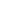 of all pairs, which determines the specificity of the prediction. Compare with Table 1.(XLS)

Dataset S6
**List of all predicted coevolving pairs in the ABC-C family.** Each Excel sheet lists the predicted coevolving pairs (including those not in structural contact) for a given fraction 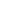 of all pairs, which determines the specificity of the prediction. Compare with Table 2 and 3.(XLS)
