# Supplementary figures and images for: Integrated Analysis of Residue Coevolution and Protein Structure in ABC Transporters
Source: PLoS One. 2012 May 8;7(5):e36546. doi: 10.1371/journal.pone.0036546 (PMC3348156; doi:10.1371/journal.pone.0036546)

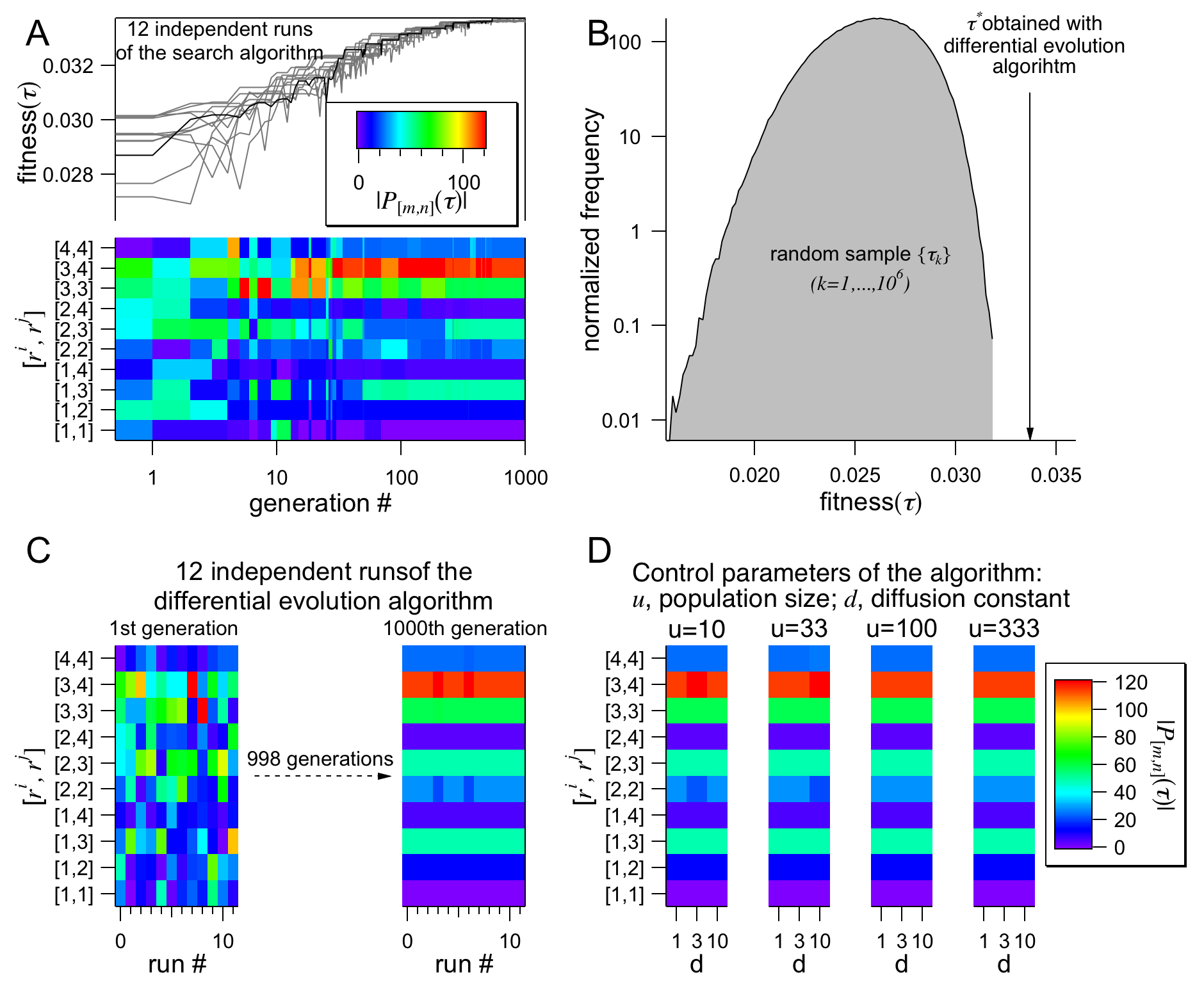

Supplement: Figure S1 — Optimization with a differential evolution algorithm. The figure shows independent runs, under various conditions defined by the control parameters of the algorithm, of the search algorithm for the optimal set of thresholds used by some coevolution detector. is defined as , where each is the coevolution threshold (eq. 11) corresponding to substitution rate class (eq. 22). Note that and so is a subset of parameters for coevolution prediction and is therefore not to be confused with the set of control parameters. The overall conclusion from this figure is that the solution identified by this heuristic algorithm is a good approximation of the global optimum. (A) The algorithm was run independently with the same control parameters as those used for the predicted pairs presented in Table 1, 2, 3. Each run was terminated at the 1000th generation (i.e. iteration). Top graph: improvement of population fitness (defined in Algorithm 1 of Text S1) in all 12 runs. The rate of improvement declined after a few hundred generations suggesting that 1000 generations are sufficient. Bottom: the evolution of is shown for one of the 12 runs (identified by black color in top graph). is the set of predicted coevolving pairs in class and so this graph further supports the previous conclusion from the top graph. (B) The approximate appears to lie close to the true optimum since , where is a random sample of size . (C) 1st generation (left): each of the 12 independent run was initialized from a distinct, randomly chosen, position of the parameter space. 1000th generation (right): all runs converge to nearly the same , indicated by . This suggests that the solution is robust against the randomness inherent to the initialization of the algorithm. (D) The solution appeared to be robust against also the control parameters of the algorithm. (TIF) [file pone.0036546.s001.tif]
