# Supplementary material for: Integrated Analysis of Residue Coevolution and Protein Structure in ABC Transporters
Source: PLoS One. 2012 May 8;7(5):e36546. doi: 10.1371/journal.pone.0036546 (PMC3348156; doi:10.1371/journal.pone.0036546)
Supplement: Text S1 — Heuristic search strategy for the optimal parameter set . The text describes a stepwise strategy for obtaining an approximate . The differential evolution search algorithm of the last step is presented as pseudocode. (PDF) [file pone.0036546.s015.pdf]

Text S1: Heuristic Search Strategy for the Optimal Parameter Set θ^*

Supporting the manuscript *Integrated Analysis of Residue Coevolution and Protein Structure in ABC Transporters* by Attila Gulyás-Kovács

As in the main text and Figure 2A, let $\{X_n\}$ be the set of N coevolution detectors selected for this study and $\{C_k\}$ the set of K substitution rate classes of position pairs ($n = 1, \dots, N$ and $k = 1, \dots, K$). For a given X_n and C_k let $t_k^{X_n}$ be the adjustable threshold and $s_k^{X_n}$ the number of sequences remaining in the alignment after filtering. (Figure 2A shows a special case when $s_k \equiv s_k^{X_1} = \dots = s_k^{X_N}$.) As explained in the main text, the set of predicted pairs P_k, for some C_k, is a function of $(t_k^{X_1}, s_k^{X_1}, \dots, t_k^{X_N}, s_k^{X_N})$ in the general case when all N detectors are combined. The present optimization problem concerns the parameter set θ given by the Cartesian product

$$\theta = \prod_{n,k} (t_k^{X_n}, s_k^{X_n}), \tag{S1}$$

where n and k take values independently from each other in $\{1, \dots, N\}$ and $\{1, \dots, K\}$, respectively.

In this study $K = 10$ and $N = 11$, so the parameter space Θ has a dimension $\dim \Theta = 219$ with (and 220 without) the constraint (cf. eq. 13 of the main text) that

$$\sum_{k=1}^{K} p_k = \gamma |\Omega|, \tag{S2}$$

where $p_k \equiv |P_k|$ is the number of predicted pairs in class C_k, and γ and Ω have the same meaning as in the main text.

The present heuristic strategy searches for the optimal θ^* in multiple steps. In each step some parameters are fixed in order to reduce $\dim \Theta$.

Before the optimization steps are discussed a few definitions and notational conventions need to be introduced. Given class C_k, the set S of structural contact pairs and the set B of structurally distant pairs, the following definitions are made:

$$b_k(\theta) = |B \cap P_k(\theta)| \tag{S3}$$

$$\rho_k^{\mathrm{FP}}(\theta) = \frac{b_k(\theta)}{|B \cap C_k|} \tag{S4}$$

$$\rho_k^{\mathrm{TP}}(\theta) = \frac{|S \cap P_k(\theta)|}{|S \cap C_k|} \tag{S5}$$

$$A_k(\alpha_k, \theta) = \int_0^{\alpha_k} \rho_k^{TP}(\theta) \mathrm{d}\rho_k^{FP}(\theta). \tag{S6}$$

(Like α in the main text, $\alpha_k \in [0, 1]$.) Thus $\rho_k^{\mathrm{TP}}, \rho_k^{\mathrm{FP}}$ and A_k are the class-specific versions of $\rho^{\mathrm{TP}}, \rho^{\mathrm{TP}}$ and A (eq. 16-18 of the main text). As eq. S4-S6 show, these quantities are functions of the parameter set θ. From this point on the notation $A_k(\alpha_k, x) \equiv A_k(\alpha_k, \theta)$ will express that only some subset $x \subset \theta$ of the parameters are varied while all other parameters are fixed. $\rho^{\mathrm{TP}}(x)$ and $\rho^{\mathrm{FP}}(x)$ have analogous meanings.

Step I and II

In step I the optimal solution $[s_k^{X_n}]^*$ for each C_k and X_n is obtained as

$$[s_k^{X_n}]^* = \arg\max_{s_k^{X_n}} A(\alpha_k = 0.1, s_k^{X_n}). \tag{S7}$$

In all subsequent steps each $s_k^{X_n}$ is held fixed at $[s_k^{X_n}]^*$. This step reduces $\dim \Theta$ to 109.

In step II all but the two best performing detectors are discarded, further reducing $\dim \Theta$ to 19. In the present study this operation is justified by the result that the two best performing detectors, CoMap and MIp, in general greatly outperformed all other detectors (Figure S5-9). However in this general discussion the two best performing detectors are denoted as X_1 and X_2 and their combination as $X_1 \wedge X_2$.

Step III

The remaining set of 20 parameters is $\{(t_k^{X_1}, t_k^{X_2})\}$ ($k = 1, \ldots, K$ and $n = 1, 2$). This set corresponds to 19 free parameters, since the constraint in eq. S2 still stands. Write $\mathbf{t}_k \equiv (t_k^{X_1}, t_k^{X_2})$. For each k fix b_k and allow $\mathbf{t}_k$ to vary. Note that it is possible not to alter b_k if $t_k^{X_1}$ and $t_k^{X_2}$ shift in the opposite direction (Figure 2A). For a given b_k define the optimal solution $\mathbf{t}_k^\circ$ as

$$\mathbf{t}_k^\circ = \arg\max_{\mathbf{t}_k} \rho_k^{\mathrm{TP}}(\mathbf{t}_k). \tag{S8}$$

Note that $\mathbf{t}_k^\circ$ is a function of b_k. But $b_k = \rho_k^{\mathrm{FP}} |B \cap C_k|$ (eq. S4) and so $\mathbf{t}_k^\circ$ can also be considered as a function of the false positive rate ρ_k^{FP}. This implies that eq. S8 can be reformulated as

$$\mathbf{t}_k^\circ(\alpha_k) = \arg\max_{\mathbf{t}_k} A_k(\alpha_k, \mathbf{t}_k), \tag{S9}$$

which is consistent with the definition of θ^* by eq. 19 of the main text.

Also note that step II-III correspond to the notion of *detector weighting*, introduced in the main text.

Step IV

Step III resulted in $\tau^\circ \equiv (\mathbf{t}_1^\circ, \ldots, \mathbf{t}_K^\circ)$, where $k = 1, \ldots, K$ and $K = 10$. As mentioned above, each $\mathbf{t}_k^\circ$ is a function of b_k and so τ° is also a function of the vector $(b_1, \ldots, b_k)$ (it might be of interest that both functions are bijective). Therefore the constraint expressed by eq. S2 allows τ° to vary, so τ° corresponds to a set of 9 free parameters. This is equivalent to *class weighting*, which was introduced in the main text.

For each $\gamma \in [0, 1]$ (eq. S2) the new framework defines the optimal parameter set $\tau^* \equiv (\mathbf{t}_1^*, \ldots, \mathbf{t}_K^*)$ as

$$\tau^* = \arg\max_{\tau^\circ} A(\alpha = \phi(\gamma), \tau^\circ), \tag{S10}$$

where ϕ is a relation transforming γ to α (cf.eq. 19 of the main text).

Writing $\mathbf{s}^* = ([s_1^{X_1}]^*, [s_1^{X_2}]^*, \ldots, [s_K^{X_1}]^*, [s_K^{X_2}]^*)$ and $\theta^* = (\tau^*, \mathbf{s}^*)$ completes the optimization process.

Implementation of Step IV

For steps I-III it is straight forward to find an efficient search algorithm for the global solutions (τ° and $\mathbf{s}^*$) but for step IV a heuristic approach was taken since this step involves 9 free parameters. Thus Eq. S10 was implemented as a slightly modified version of the differential evolution algorithm described on page 149 of Feoktistov V (2006) Differential Evolution, volume 5 of Springer Optimization and Its Applications. Springer US. Appendix A. This modified algorithm is presented below. To simplify notation the following conventions are introduced: $\mathbf{t}_k^i \equiv [\mathbf{t}_k^\circ]^i$ and $\tau \equiv \tau^\circ$. These conventions also apply to Figure S1, which illustrates some properties of the algorithm.

Algorithm 1: Optimization with differential evolution

Input: γ, fraction of predicted pairs in $|\Omega|$ total pairs

`// control input parameters`

Input: u, population size

Input: d, diffusion constant

Input: g, number of generations

Data: $U = \{\tau^1, \tau^2, \cdots, \tau^u\}$, population of u individuals

Data: $\tau^i = (\mathbf{t}_1^i, \ldots, \mathbf{t}_K^i)$, each individual τ^i is a parameter set containing K thresholds $\mathbf{t}_k^i \equiv [\mathbf{t}_k^\circ]^i$, where for each i the definition of $[\mathbf{t}_k^\circ]^i$ is given by eq. S8 and K is the number of substitution rate classes.

Data: τ^{trial}, depending on its fitness, the trial individual may change the population in each generation.

Function: `CreateRandomIndividual`(Constraint), to initialize the population

Function: `Fitness`$(\tau^i) = \rho^{\text{TP}}(\tau^i)$, where $\rho^{\text{TP}}(\tau^i)$ is the true positive rate (eq. 16 of the main text)

Function: `CreateTrialIndividual`(τ^i, U, d), creates a trial individual from τ^i, by a mutation and a compensatory mutation, based on 3 other individuals of the population. See Algorithm 2 for details.

Output: τ^*, fittest individual (optimized parameter set)

`// initialize population`

`// the number of all predicted pairs` $|P| \equiv \sum_k |P_k(\mathbf{t}_k)|$ `is constrained (eq. S2)`

Constraint: $|P_k(\mathbf{t}_k)| = \text{round}(\gamma \times |\Omega|)$;

$\tau^1 \longleftarrow$ `CreateRandomIndividual`(Constraint) ;

$\tau^* \longleftarrow \tau^1$;

for $i = 2$ **to** u **do**

    $\tau^i \longleftarrow$ `CreateRandomIndividual`(Constraint) ;

    if `Fitness`$(\tau^i) >$ `Fitness`(τ^*) **then**

        $\tau^* \longleftarrow \tau^i$;

    end

end

`// evolve population`

for $l = 1$ **to** g **do**

    for $i = 1$ **to** u **do**

        $\tau^{\text{trial}} \longleftarrow$ `CreateTrialIndividual`(U, τ^i, d) ;

        if `Fitness`$(\tau^{trial}) >$ `Fitness`(τ^i) **then**

            $\tau^{\text{i}} \longleftarrow \tau^{\text{trial}}$;

            if `Fitness`$(\tau^{trial}) >$ `Fitness`(τ^*) **then**

                $\tau^* \longleftarrow \tau^{\text{trial}}$;

            end

        end

    end

end

return τ^* ;

Algorithm 2: CreateTrialIndividual(U, τ^i, d)

Input: U, population

Input: $\tau^i = (\mathbf{t}_1^i, \ldots, \mathbf{t}_K^i)$, ith individual of population

Input: d, diffusion constant

Output: $\tau^{\text{trial}} = (\mathbf{t}_1^{\text{trial}}, \ldots, \mathbf{t}_K^{\text{trial}})$, trial individual

// Mutation of τ^i is based on randomly chosen individuals τ^a, τ^b, τ^c and substitution rates p, q

randomly select $\tau^a, \tau^b, \tau^c \in U$ such that $a \neq b \neq c \neq l$;

randomly select p, q such that $1 \leq p, q \leq K$ and $p \neq q$;

// The mutation uniquely determines the compensatory mutation under the constraint below

$\mathbf{t}_p^{\text{mut}} \longleftarrow \mathbf{t}_p^a + d(\mathbf{t}_p^b - \mathbf{t}_p^c)$;

// Ensure that the mutation does not affect number of all predicted pairs

Constraint: $|P_p^{\text{trial}}(\mathbf{t}_p^i)| + |P_q^{\text{trial}}(\mathbf{t}_q^i)| = |P_p^{\text{trial}}(\mathbf{t}_p^{\text{mut}})| + |P_q^{\text{trial}}(\mathbf{t}_q^{\text{mut}})|$;

$\mathbf{t}_q^{\text{mut}} \longleftarrow$ `CompensatoryMutation`$(\text{Constraint}, \mathbf{t}_p^i, \mathbf{t}_q^i, \mathbf{t}_p^{\text{mut}})$;

// The trial individual is the mutated copy of τ^i

$\tau^{\text{trial}} \longleftarrow \tau^i$;

$\mathbf{t}_p^{\text{trial}} \longleftarrow \mathbf{t}_p^{\text{mut}}$;

$\mathbf{t}_q^{\text{trial}} \longleftarrow \mathbf{t}_q^{\text{mut}}$;

return τ^{trial} ;
